# Metabonomic profiling of clubroot-susceptible and clubroot-resistant radish and the assessment of disease-resistant metabolites

**DOI:** 10.3389/fpls.2022.1037633

**Published:** 2022-12-08

**Authors:** Jingwei Li, Tingmin Huang, Jinbiao Lu, Xiuhong Xu, Wanping Zhang

**Affiliations:** ^1^ Vegetable Research Institute, Guizhou University, Guiyang, China; ^2^ College of Agriculture, Guizhou University, Guiyang, China

**Keywords:** gingerol, ginsenoside, imipenem, metabolome, *Plasmodiophora brassicae*, *Raphanus sativus*

## Abstract

*Plasmodiophora brassicae* causes a serious threat to cruciferous plants including radish (*Raphanus sativus* L.). Knowledge on the pathogenic regularity and molecular mechanism of *P. brassicae* and radish is limited, especially on the metabolism level. In the present study, clubroot-susceptible and clubroot-resistant cultivars were inoculated with *P. brassicae* Race 4, root hairs initial infection of resting spores (10^7^ CFU/mL) at 24 h post-inoculation and root galls symptom arising at cortex splitting stage were identified on both cultivars. Root samples of cortex splitting stage of two cultivars were collected and used for untargeted metabonomic analysis. We demonstrated changes in metabolite regulation and pathways during the cortex splitting stage of diseased roots between clubroot-susceptible and clubroot-resistant cultivars using untargeted metabonomic analysis. We identified a larger number of differentially regulated metabolites and heavier metabolite profile changes in the susceptible cultivar than in the resistant counterpart. The metabolites that were differentially regulated in both cultivars were mostly lipids and lipid-like molecules. Significantly regulated metabolites and pathways according to the *P* value and variable important in projection score were identified. Moreover, four compounds, including ethyl α-D-thioglucopyranoside, imipenem, ginsenoside Rg1, and 6-gingerol, were selected, and their anti-*P. brassicae* ability and effects on seedling growth were verified on the susceptible cultivar. Except for ethyl α-D-thioglucopyranoside, the remaining could inhibit clubroot development of varing degree. The use of 5 mg/L ginsenoside Rg1 + 5 mg/L 6-gingerol resulted in the lowest disease incidence and disease index among all treatments and enhanced seedling growth. The regulation of pathways or metabolites of carbapenem and ginsenoside was further explored. The results provide a preliminary understanding of the interaction between radish and *P. brassicae* at the metabolism level, as well as the development of measures for preventing clubroot.

## 1 Introduction

Radish (*Raphanus sativus* L.), which belongs to the Cruciferae family, is economically important. It is mainly cultivated to produce root vegetables, processed food raw ingredients, fodder, pigment, cosmetics, pharmaceutical materials, and bio-oil (Chen et al., 2016a; Lee and Park, 2017; Silvestre et al., 2018; Lee and Park, 2020; Silvestre et al., 2020). Pathogens, including *Plasmodiophora brassicae*, greatly affect the metabolite composition and appearance quality of radish. *P. brassicae*, a soil-borne and obligate biotrophic intracellular pathogen belonging to *Plasmodiophorales*, disrupts the root development in the host root and the formation of finger, bar, or spherical gall roots and inhibits water and mineral uptake in host plants. *P. brassicae* is responsible for causing clubroot, one of the most devastating diseases striking cruciferous crop production (Dixon, 2009; Kageyama and Asano, 2009; Yang et al., 2020). The resting spores of *P. brassicae* survive in the soil for up to 20 years (Donald and Porter, 2009; Kageyama and Asano, 2009) and is difficult to control (Kowata-Dresch and Mio, 2012; Yang et al., 2020). The studies of clubroot have been conducted frequently on various cruciferous plants but are limited in radish (Akaba et al., 2009; Kamei et al., 2010).

The “Omics” techniques have become powerful and convenient tools for plant–disease interaction studies since the global profiles of molecular and biochemical changes were revealed. A large number of studies based on transcriptomic and proteomic analyses have been conducted to uncover the basic pathology involved in the response of cruciferous crops to *P. brassicae* invasion. The comparative transcriptome technique has been widely used to gain a better understanding of the interaction between *Arabidopsis thaliana* and *P. brassicae*, which are the most frequently used materials for clubroot researches (Siemens et al., 2006; Zhao et al., 2017; Irani et al., 2018). Luo et al. (2018) offer an important public transcriptome information platform to study the mechanism by which the biocontrol strain *Zhihengliuella aestuarii* inhibited *P. brassicae* infecting *Brassica juncea*; a deeper view of drastic defense response of *B. oleracea* to spores in the secondary infection stage were offered by Ning et al. (2019) in the next year. Galindo-González et al. (2020) supplied the regularity analysis of key genes involved in salicylic acid-mediated immunity, which was stimulated by *P. brassicae* infection on *B. napus.* Gazengel et al. (2021) furnish an illustration that abiotic factors such as nitrogen supply mediated the clubroot resistance/susceptibility of *B. napus.* Interestingly. Though the spores were parasitic in roots, the number of differentially expressed genes between infected and uninfected samples was larger in shoots than in roots (Irani et al., 2018). Moreover, the deep sequencing and degradome analyses were conducted as well, Wei et al. (2016) demonstrated that seleno compound metabolism and plant hormone signal transduction were invoved in *B. pekinensis*’s reponse to *P. brassicae*. Proteomic analysis, as one of the most wildly used omics techniques, was performed to understand better the interaction mechanisms at the post-transcriptional level and to facilitate the identification of key molecular determinants (Moon et al., 2020). Proteins participated in hormone metabolism pathways [jasmonic acid (JA)/ethylene-mediated systemic resistance and cytokinin metabolism], pathogenesis-related protein, plant–pathogen interaction signaling pathways, lignin biosynthesis, glycolysis, intracellular calcium homeostasis, and detoxification of reactive oxygen species (ROS) were differentially regulated in clubroot-diseased crops (Cao et al., 2008; Ji et al., 2018). Clubroot resistance genes are frequently employed in *P. brassicae*-resistant cultivar breeding (Yang et al., 2020). The data of shotgun label-free proteomic analysis revealed that CR genes triggered the distinguishing of *P. brassicae* invading and the activation of defense responses in clubroot-resistant *B. rapa via* a unique signaling pathway distinct from common modes of recognition receptors reported in many other plant–pathogen interactions (Song et al., 2016). In *B. oleracea* plants, abscisic acid-responsive protein, fructose-bisphosphate aldolase, and glucose sensor interaction protein might mediate basal defense against *P. brassicae* in the resistant cultivar; while in sensitive genotype, cobalamin-independent methionine synthase was significantly regulated and might be a biomarker for the susceptibility of the host (Moon et al., 2020).

Metabolite are the ultimate form of gene expression. More recently, the rapid development of high-pressure liquid chromatography (HPLC), high-resolution mass spectrometry (MS), nuclear magnetic resonance spectroscopy (NMR) technologies and the computer-driven analytical and data mining packages, allows people to identify and quantify individual metabolites and conduct a nontarget metabonomic assay (Jorge et al., 2016; Subbaraj et al., 2019). Compared with other techniques, metabonomics is not dependent on the availability of published specific genome information for data analysis and processing (Jorge et al., 2016). Moreover, it provides the global profile of the results of all the biochemical processes and their products, thus carrying an imprint of all genetic, epigenetic, and environmental factors, which constructs a bridge between genotypes and phenotypes (Jorge et al., 2016; Krumsiek et al., 2016; Barrett et al., 2021). Although the metabolome has been widely applied to reveal plant biochemical responses to stresses including plant pathogens (Hartley and Gange, 2009; Lazebnik et al., 2014; Mareya et al., 2020; Sanmartín et al., 2020), limited studies has been conducted on *P. brassicae* pathology (Pedras et al., 2008; Hirani and Li, 2015; Song et al., 2015).

A large number of clubroot pathology studies have been conducted on cruciferous crops other than radish, more especially, metabonomics resreaches are limited. It is expected that host metabolite components vary during disease outbreak. The occurrence regularity of clubroot induced by the artificial inoculation of *P. brassicae* Race 4 was compared between susceptible and resistant genotypes. The diseased seedlings of two cultivars in the gall developing stage were used in combination with an liquid chromatography-mass spectrum in series (LC-MS/MS) platform for metabonomic analysis. The measurement of the metabolites dynamic changes would reflect the regularity of susceptible and resistant radish cultivars responding to *P. brassicae* infection on the global metabolic level. Finally, the anti-*P. brassicae* functions of differential regulated metabolites were tested. The findings help in selecting the biocontrol metabolites for clubroot disease and might provide metabolite targets for resistant cultivar breeding.

## 2 Materials and methods

### 2.1 Materials

*Raphanus sativus* “Jiangnanyuanbai” (“JNYB”) from China, chosen for its high susceptibility, and “Daehanbaekchun” (“DHBC”) from Korea, chosen for its resistance, were selected from more than 97 commercial cultivars in *P. brassicae–*contaminated radish farm in Weining county, Bijie City, Guizhou province, China (**Supplementary Figure S1** and **Table 1**). The resting spores of *P. brassicae* Race 4 (shortened as P4) were purified from diseased plants and soil from the same farm and identified using the William differential system. The spores were inoculated on JNYB roots, the diseased roots were conserved at –20°C until use.

**Table 1:** Assessment of cultivar resistance to *P. brassicae* Race 4 in different growth stages.

	Clubroot incidence (%)		
Cultivar	Pre-cortex splitting stage	Cortex splitting stage	Post- cortex splitting stage
“JNYB”	3.0 ± 1.4 c *	10.3 ± 1.9 b *	84.7 ± 5.9 a *
“DHBC”	0 ± 0 b	0.9 ± 1.4 b	3.8 ± 1.1 a
	Clubroot index		
Cultivar	Pre-cortex splitting stage	Cortex splitting stage	Post- cortex splitting stage
“JNYB”	6.5 ± 1.9 c *	18.5 ± 3.7 b *	43.9 ± 5.6 a *
“DHBC”	0 ± 0 c	2.8 ± 0.9 b	7.1 ± 1.1 a

A total of 20 randomly selected samples were treated and measured in each replication, 3 replications were conducted. Data were presented by mean value ± standard error (SE). Letters or * indicates significant differences among each development stages or between two cultivars respectively. Significant differences were analyzed by one-way ANOVA and Student’s t test on P < 0.05. The disease incidence and index of mock of both cultivars were 0.

### 2.2 *P. brassicae* inoculation and plant incubation

The extraction and purification of P4 spores were conducted as previously described by Arif et al. (2021) with slight modification. Frozen mature galls of “JNYB” radish were thawed under room temperature and homogenized in a blender. The resulting slurries were filtered through gauze. Finally, the spores were obtained after centrifugation of the filtrate at 3000 rpm for 15 min. The spore concentration was determined with ddH_2_O using a hemocytometer. The spore suspension was diluted in 1/4 Hoagland to a concentration of  10^7^ CFU/mL solution. The pH was adjusted to 5.0 with 1M sodium citrate aqueous solution. Further, spore suspension was used for hydroponic and substrate (peat:perlite:vermiculite = 1:1:1) cultivation respectively.

**Figure 1:**
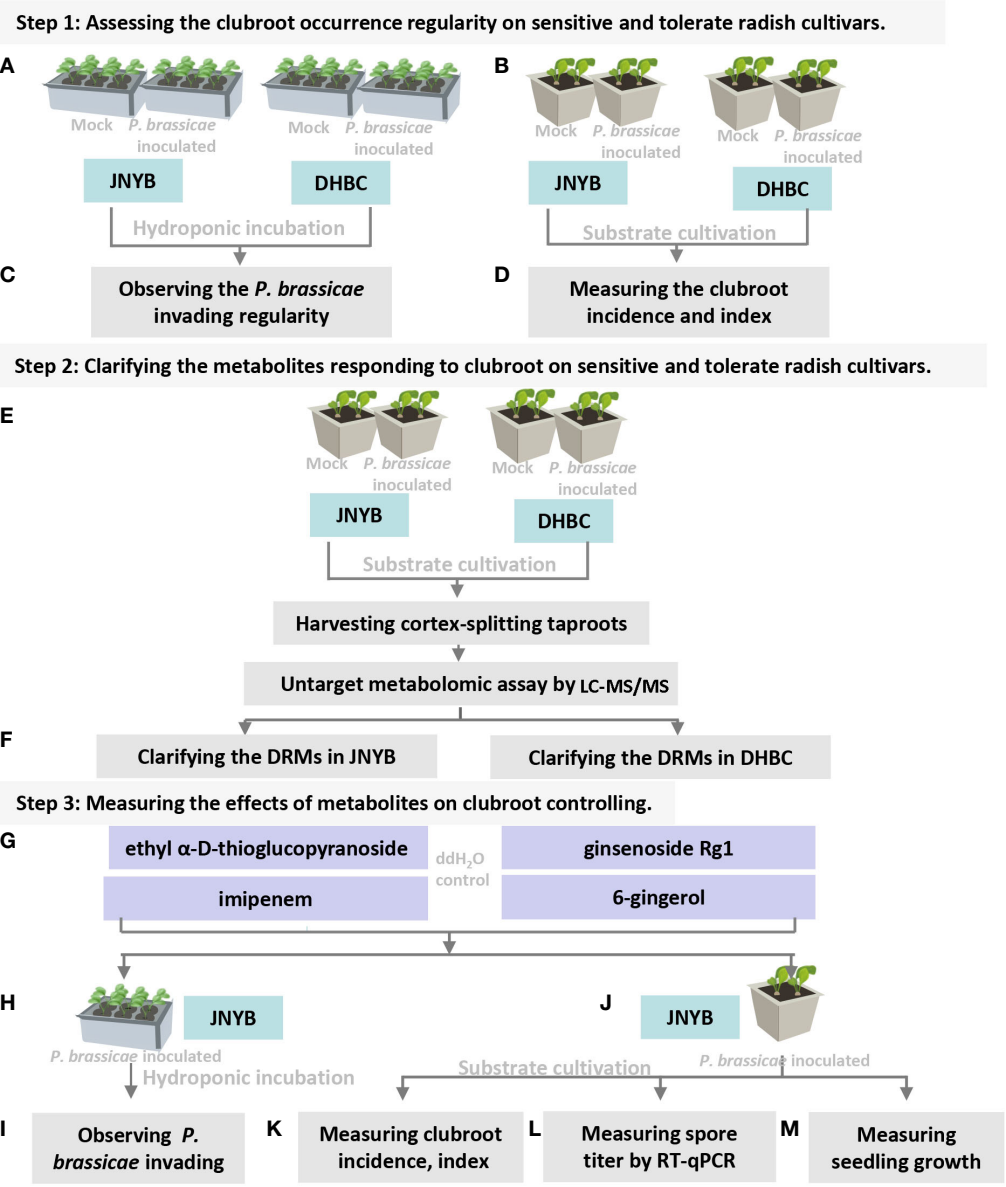
Flowchart of present study. **(A)** Inoculation of *P. brassicea* spore to hydroponic cultured “JNYB” and “DHBC”. **(B)** Inoculation of *P. brassicea* spore to substrate cultivated “JNYB” and “DHBC”. **(C)** Observation of *P. brassicea* invading to root hairs of two radish cultivars. **(D)** Clubroot disease incidence and index measurement of two radish cultivars. **(E)** Inoculation of *P. brassicea* spore to substrate cultivated “JNYB” and “DHBC”, collecting cortex-splitting root samples for LC-MS/MS measurement. **(F)** Clarification of differentially regulated metabolites in two cultivars. **(G)** Four differentially regulated metabolites were selected for the following studies. **(H, J)** Application of selected metabolites in hydroponic and substrated cultivation of “JNYB” radish. **(I)** Observation of metabolites’ effects on *P. brassicea* invading to root hairs. **(K–M)** Mearument of metabolites’ effects on clubroot diseased severity, *P. brassicea* proliferation, and radish growth. The ddH2O treatment was used as control in this study.

The seeds of “JNYB” and “DHBC” were surface sterilized in 1% sodium hypochlorite for 5 min and rinsed thrice with ddH_2_O. The sterilized seeds were sown in plastic trays containing a wet filter paper and maintained at 28°C (daytime)/15°C (nighttime) under a 16-h/8-h light/dark cycle with 80% relative humidity. For hydroponic incubation, 10 of 7-day-old uniform seedlings of the sensitive cultivar “JNYB” and resistant cultivar “DHBC” were incubated in 4 L spore suspension (**Figure 1A**) for spore invading observation, and at least 80 seedlings were incubated. The seedlings were also transplanted into pots containing 1 L substrate contaminated with 30 mL of 10^7^ CFU/mL P4 spores (**Figure 1B**) to see the disease occurrence regularity, and at least 80 seedlings were incubated. Control were those samples incubated with only 1/4 Hoagland supplemented hydroponic and substrate culture (mock, **Figures 1A, B**). All seedlings were incubated in the same environment as described earlier for seed germination with 60% relative humidity and a light intensity of 45 μmol s^−1^ m^−2^ provided using cool-white fluorescent tubes. Seedlings were watered every 3 days and 1/4 Hoagland solution was given every two weeks during the culture.

### 2.3 Measurement of spore invading, disease incidence and index

For hydroponic incubation, 7-days treated roots were fixed in 70% FAA solution [3.7% paraformaldehyde (*w*/*v*), 5% acetic acid (*v*/*v*), and 50% ethanol (*v*/*v*)] for 24 h, stained with safranine T for 10 min, and rinsed with ddH_2_O thrice. The middle pieces of stained roots were observed under a microscope (BX53, Olympus, Japan) to analyze the situation in which resting spores concentrated on radish roots after co-incubation with *P. brassicae*. 20–30 seedlings were involved in each treatment, and 3 repeats were conducted (**Figure 1C**). The life cycle of *P. brassicae* were described as Liu et al. (2020) recorded. The disease incidence and disease index were observed every 5 days after planting in substrate. Especially, The root samples in the stages of pre-cortex splitting, cortex splitting, and post-cortex splitting (expanding stage) (Yu et al., 2016a; Yu et al., 2016b) were harvested and measured. At least 20 plants in each stage were randomly sampled. The experiments were repeated three independent times (**Figure 1C**). The number of symptomatic plants was determined, and the disease incidence and disease index were calculated according to Horikoshi (2002) with some modification. Incidence rate (%) = Number of diseased plants/Total number of investigated plants × 100. The roots in each stage were washed to determine disease severity. The disease severity was classified into six categories based on the following criteria; not clubbed, category 0; less than 30% of lateral roots clubbed, category 1; less than half of tap roots clubbed and 50% of lateral roots clubbed, category 3; half of the tap roots clubbed and 75% of lateral roots clubbed, category 5; more than half of tap roots clubbed and more than 90% of the lateral roots clubbed, category 7; and more than 75% of tap roots clubbed and lateral roots clubbed, category 9. Disease index = [Σ (Number of diseased plants in each stage × Relative value of category)]/(Total number of plants under investigation × Highest incidence of disease) × 100 (**Figure 1D**).

### 2.4 Nontargeted metabonomic assay

#### 2.4.1 Material sampling and metabolite extraction

Four kinds of samples, 1/4 Hoagland solution treated mock and P4 suspension (10^7^ CFU/mL) treated cortex-splitting tap roots of “JNYB” and “DHBC” from substrate cultivation, were collected and washed under flowing water for 3 h and used for metabonomic assay (**Figure 1E**). The seeds of two cultivars were sowed on the same date, considering that the seedlings of “JNYB” developed faster than that of “DHBC”, mock and treated root samples of “JNYB” were sampled, processed, and analyzed earlier than that of “DHBC”, thus the metabonomic assay and analysis were conducted separately on two cultivars. 60 roots were randomly harvested, 10 roots of each treatment were merged in a single sample and smashed using liquid nitrogen, six repeats were conducted. The metabolites were extracted with 120 μL of precooled 50% methanol buffer from 100 mg sample. The mixture of metabolites was vortexed for 1 min, incubated for 10 min at room temperature, and then stored at –20°C overnight. The mixture was centrifugated at 4000 rpm for 20–25 min, and the supernatant was subsequently transferred to 96-well microplates. The samples were stored at -80°C prior to the LC-MS/MS analysis. Pooled quality control (QC) samples were prepared by combining 10 μL of each extraction mixture as well to evaluation and correction of signal drift, which is beneficial to improve the quality of inter batch experimental data.

#### 2.4.2 LC-MS/MS analysis

The samples were analyzed using a TripleTOF 5600 Plus tandem mass spectrometer (SCIEX, Warrington, UK) with both positive and negative ion modes. Chromatographic separation was performed using an ultra-high-performance liquid chromatography (UPLC) system (SCIEX). An ACQUITY UPLC T3 column (100 mm × 2.1 mm, 1.8 μm, Waters, UK) was used. It was introduced to separate metabolites; the mobile phase consisted of solvent A (water and 0.1% formic acid) and solvent B (acetonitrile and 0.1% formic acid). The gradient elution conditions were as follows with a flow rate of 0.4 mL/min: 5% solvent B for 0–0.5 min; 5%–100% solvent B for 0.5–7 min; 100% solvent B for 7–8 min; 100%–5% solvent B for 8–8.1 min; and 5% solvent B for 8.1–10 min. The column temperature was maintained at 35°C. The TripleTOF 5600 Plus system was used to detect metabolites eluted from the column. The curtain gas pressure was set at 30 pounds per square inch (PSI); the ion source gas1 and gas2 pressure was set at 60 PSI. The interface heater temperature was 650°C. The ion spray floating voltage was set at 5 kV for the positive ion mode, while it was set at –4.5 kV for the negative ion mode. The MS data were acquired in the information dependent acquisition (IDA) mode. The four time bins were summed for each scan at a pulse frequency of 11 kHz by monitoring the 40-GHz multichannel TDC detector with four‐anode/channel detection. Dynamic exclusion was set for 4 s. During the entire acquisition period, the mass accuracy was calibrated for every 20 samples. QC sample was analyzed every 10 samples to evaluate the stability of the LC-MS/MS. Finally, a merge analysis of positive and negative ion modes was conducted.

#### 2.4.3 Metabonomics data processing

The acquired LC-MS/MS raw data files were converted into mzXML format and then processed using the XCMS, CAMERA, and metaX toolbox included in R software. Each ion was identified by the comprehensive information of retention time and *m*/*z*. The intensity of each peak was recorded, and a three-dimensional matrix containing arbitrarily assigned peak indices (retention time–*m*/*z* pairs), sample names (observations), and ion intensity information (variables) was generated. Data preprocessing method are described in the literature (Pu et al., 2021). Briefly, the primary identification of metabolites was carried out using the Human Metabolome Database (HMDB) and Kyoto Encyclopedia of Genes and Genomes database (KEGG). The secondary fragment data in MS were matched with the secondary library of in-house metabolite standard (established by Huangzhou LC-bio Co., Ltd) (Tang et al., 2022). The peak intensity data were further preprocessed using metaX toolbox. Features detected in <50% of QC samples or 80% of test samples were removed, and the values for missing peaks were extrapolated with the k-nearest neighbor algorithm to further improve the data quality. Principal component analysis (PCA) was performed to detect outliers and batch effects using the pre-processed dataset, data analysis was conducted by R stats package prcomp function, graphics were drawn by R ggplot2 package stat_ellipse function. QC-based robust LOESS signal correction was fitted to the QC data with respect to the order of injection to minimize the signal intensity drift over time. In addition, the relative standard deviations of the metabolic features were calculated across all QC samples, and those with standard deviations >30% were removed. The group datasets were normalized before the analysis was performed. Data normalization was performed on all samples using the probabilistic quotient normalization algorithm. Then, QC-robust spline batch correction was performed using QC samples. The *P* value analyzed by the Student’s *t* test, which was then adjusted for multiple tests using an false discovery rate (FDR) (Benjamini–Hochberg), was used for different metabolite selection. We also conducted the supervised partial least squares discriminant analysis (PLS-DA) using metaX toolbox on variables by the discriminant profiling statistical method to identify more specific differences between the groups according to Tang et al. (2022). The variable important in projection(VIP) cutoff value of 1.0 was set to select important features. The differentially expressed metabolites (DRMs) between mock and diseased taproots of “JNYB” or “DHBC” were analyzed respectively to reduce the influence of genetic background to the present study (**Figure 1F**). The datasets presented in this study can be found in online repositories. The names of the repository/repositories and accession number(s) can be found below: MetaboLights, accession numbers MTBLS6347 and MTBLS6348.

### 2.5 Assessment of DRMs’ effects on clubroot inbihition

#### 2.5.1 Preparation of metabolite solution

Glucosinolate and carbapenem were selected from “JNYB” DRMs or differentially regulated pathways, ginsenoside and gingerol were detected from “DHBC”. Standard samples of ethyl α-D-thioglucopyranoside (shortened as EDT, representing glucosinolate, HPLC ≥ 98%, Maikelin Bio, Shanghai, China), imipenem (IPM, representing for carbapenem, HPLC ≥ 98.5%, Dalian, China), ginsenoside Rg1 (GR, HPLC ≥ 98%, Nakeli Bio, Chengdu, China), and 6-gingerol (6G, HPLC ≥ 98%, Shengqing Bio, Xi’an, China) were purchased. Further, 2.5 mg/L and 5 mg/L of EDT, GR, and 6G (shortened as 2.5 or 5 EDT, GR, and 6G), and 10 mg/L and 20 mg/L of IPM (10 and 20 IPM), were made using 1/4 Hoagland solution. Meanwhile, 5 mg/L EDT + 20 mg/L IPM (EDT + IPM), 5 mg/L EDT + 5 mg/L GR (EDT + GR), 5 mg/L EDT + 5 mg/L 6G (EDT + 6G), 20 mg/L IPM + 5 mg/L GR (IPM + GR), 20 mg/L IPM + 5 mg/L 6G (IPM + 6G), 5 mg/L GR +5 mg/L 6G (GR + 6G), 5 mg/L EDT + 20 mg/L IPM + 5 mg/L GR (EDT+ IPM +GR), 5 mg/L EDT + 20 mg/L IPM + 5 mg/L 6G (EDT + IPM + 6G), 5 mg/L EDT + 5 mg/L GR + 5 mg/L 6G (EDT + GR + 6G), 20 mg/L IPM + 5 mg/L GR + 5 mg/L 6G (IPM + GR + 6G), and 5 mg/L EDT + 20 mg/L IPM + 5 mg/L GR + 5 mg/L 6G (EDT + IPM + GR + 6G) were also configured using 1/4 Hoagland solution. Same volume of 1/4 Hoagland solution treated samples were used as mock (**Figure 1G**). The pH of all metabolite solutions was adjusted to 5.0 with 1M sodium citrate aqueous solution.

#### 2.5.2 Assessment of metabolites by hydroponic culture and substrate cultivation

For this, metabolite solution with 10^7^ CFU/mL P4 spores were made for hydroponic culture (**Figure 1H**). The seedling incubation and root observation were performed as previously described (**Figure 1I**). The seeds of “JNYB” were germinated and planted in a P4-contaminated substrate as earlier described (**Figure 1J**). Then, 20 mL of metabolite solutions were applied by substrate irrigation after seedling planting. The metabolite treatments were conducted every 10 days and thrice. The roots in the cortex-splitting stage were collected, and the disease incidence and disease index were determined as earlier (**Figure 1K**). At least 20 plants in each stage were randomly sampled. The experiments were repeated thrice.

#### 2.5.3 *P. brassicae* titer determination in metabolite-treated roots

The contents of *P. brassicae* in roots were detected using Real-time quantitative polymerase chain reaction (RT-qPCR). Total RNA was extracted from the root samples of metabolite treatments and control using an RNA prep Pure Plant Kit (Tiangen, Beijing, China). Total RNA (200 ng) was reverse transcribed using an ReverTra Ace qPCR RT Kit (with gDNA remover) (Toyobo, Osaka, Japan). The qPCR analysis was performed using a LightCycler FastStart DNA Master SYBR Green I kit (Roche, Basel, Switzerland) on an FQD-96A Real-Time PCR detection system (BIOER, Huangzhou, China). According to *P. brassicae* ITS(internal transcribed spacer) sequence in genome (access number GCA_001049375.1), primer design using software Primer 5.0, the primers sequences were PB-F: 5’-CACACTGTTAAATTGCGGAC-3’ and PB-R: 5’-ACAGACGACTATACTTACGAC-3’. 30 roots were sampled, 10 roots in each treatment were merged as one sample, and 3 independent biological replicates were used for each analysis with at least 3 technical replicates each. Actin gene (XM_018620829) was used as the internal control. The primer sequences were as follows: AT-F: 5’-GCATCACACTTTCTACAAC-3’, AT-R: 5’-CCTGGATAGCAACATACAT-3’. The comparative 2^-ΔΔCT^ method was used to calculate the relative gene expression (Livak and Schmittgen 2001) (**Figure 1L**).

#### 2.5.4 Assessment of the effects of metabolites on seedling morphology

The seeds were sowed in the substrate with P4, and the metabolites were applied as decribed above. 1/4 Hoagland solution was used as control. The morphological indexes of seedlings were recorded by measuring fresh weight (FW), number of true leaves, stem height, and diameter 30 days after metabolite treatment. Seedlings were watered every 3 days and 1/4 Hoagland solution was given every two weeks during the culture. At least 10 randomly selected samples were measured in each replication, 3 independent biological replicates were used (**Figure 1M**).

### 2.6 Statistical analysis

The data were analyzed using One-way ANOVA analysis of variance. The least significant difference was calculated at *P* < 0.05.

## 3 Results

### 3.1 Infection of *P. brassicae* and the development of root galls

**Figure 2:**
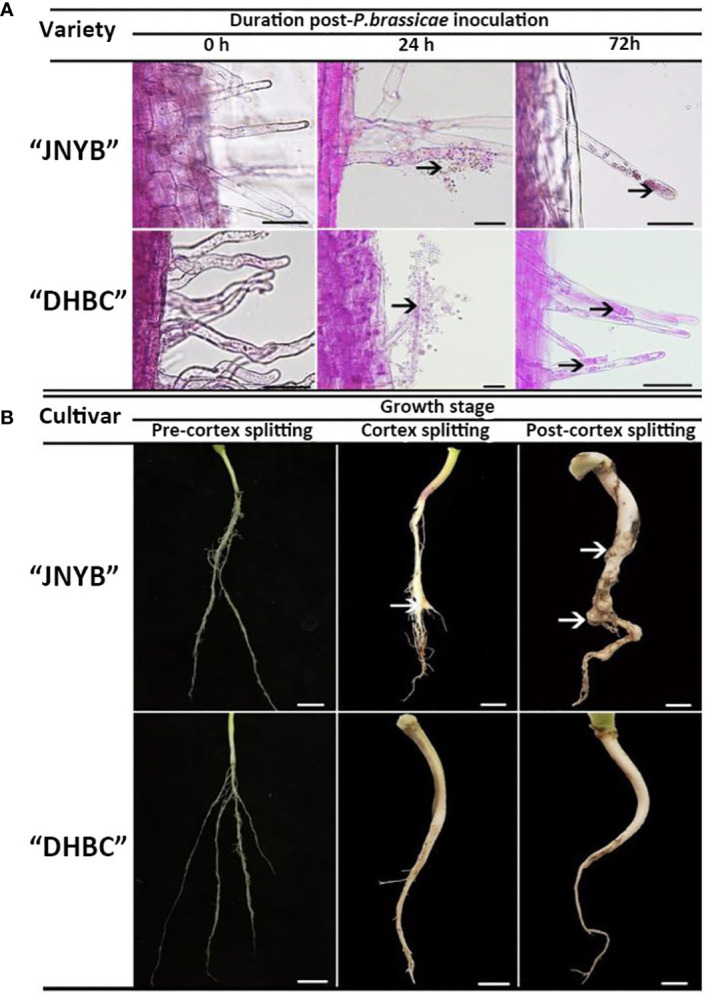
Growth of *P. brassicae* within the root hair of radish 0, 24, and 72 h after inoculation with resting spores **(A)** and the clubroot disease incidence in radish tap root in the pre-cortex splitting, cortex splitting, and post-cortex splitting stages **(B)** Black arrows in **(A)** indicate spores and zoosporangia., White arrows in **(B)** indicate swelling roots. Scale bar in **(A)**, **(B)** equals to 100 μm and 1 cm, respectively.

The dual culture of root hairs and *P. brassicae* under hydroponic culture conditions was conducted for the serial observation of root hair infection by resting spores. Compared with the mock, the encysted zoospores root hair infections started after 24 h of inoculation. At this point, the encysted zoospores accumulated in the surrounding of the root hairs and some of them invaded the roots and became primary plasmodia. The multinucleate zoosporangial plasmodium expanded to fill the terminal of the root hair after spore infection and the zoospore release from multinucleate zoosporangial plasmodium in a single root hair in radish root hair culture was serially observed for 72 h after inoculation. Interestingly, no significant differences were observed on both “JNYB” and “DHBC”, which exhibited totally different susceptibility to *P. brassicae* (**Figure 2A**).

Significant differences in clubroot resistance were detected between “JNYB” and “DHBC” seedlings (**Supplementary Figure S1** and **Table 1**); however, the Incidence regularity of root galls on both genotypes were similar. In the pre-cortex-splitting stage, rare swelling roots were observed on “JNYB”. Further, 3% of the inoculated “JNYB” seedlings exhibited root galls, and the average disease index stayed at a low level of 6.5. The roots of “DHBC” were totally healthy. The primary intensive appearance of root clubs was observed in the cortex-splitting stage. The disease incidence (10.3%) and disease index (18.5) of “JNYB” tap roots were significantly higher than earlier. Moreover, the diseased seedlings and root galls appeared at this stage on “DHBC”. The disease incidence and disease index were 0.9% and 2.8, respectively. In the post-cortex splitting stage, highly developed and diseased tap roots were assessed; 84.7% of “JNYB” seedlings were diseased, and the disease index was as high as 43.9. Compared with the sensitive genotype, a limited number of roots were diseased, with a disease incidence of 3.8% and disease index of 7.1. The infection ratio and severity were significantly lower than those of “JNYB” in the same developmental stage of “DHBC”, but still, significantly higher than those in the cortex splitting stage (**Figure 2B**, **Supplementary Figure S2** and **Table 1**).

### 3.2 Metabolic characteristics of radish response to *P. brassicae* infection

**Figure 3:**
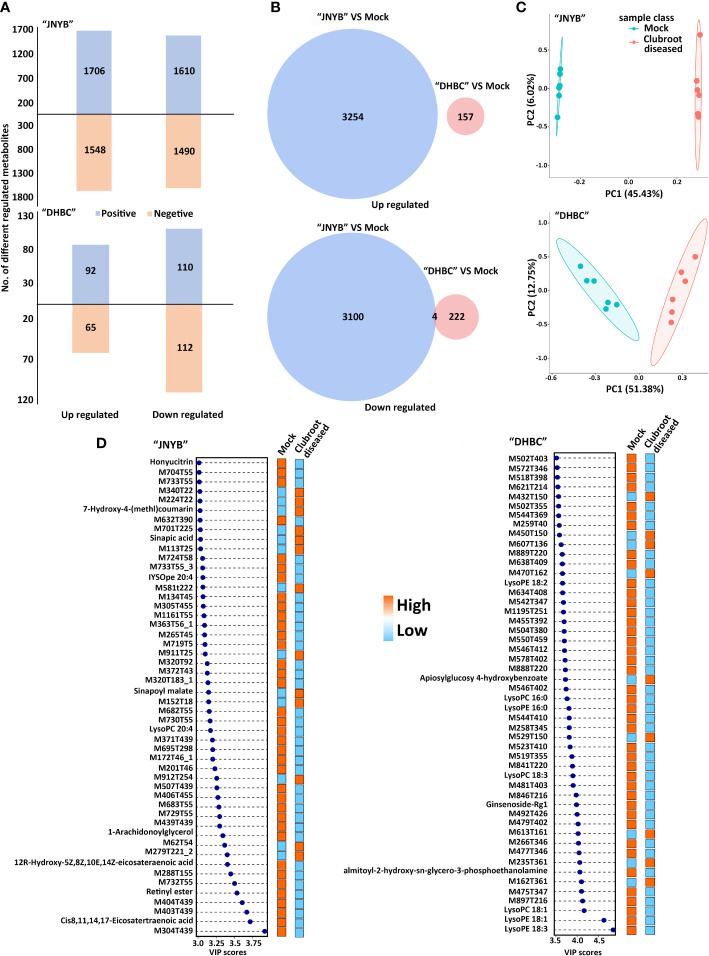
Analysis of DRMs between *P. brassicae*-susceptible and *P. brassicae*-resistant cultivars upon clubroot disease infection. **(A)** Number of up- and downregulated DRMs between “JNYB” and “DHBC” in positive and negative ion modes. **(B)** Overlap among clubroot disease-responsive metabolites between two cultivars. **(C)** PCA of metabolic profiles of “JNYB” and “DHBC” under control and clubroot disease. **(D)** Fifty top metabolites according to the VIP score of two cultivars in response to clubroot. Positive and negative in A refers to positive ion mode and negative ion mode, respectively.

The plant samples collected in the cortex splitting stage (root gall developing stage) were analyzed for each genotype using the global untargeted metabonomic analysis based on the LC-MS/MS platform. The growth and developmental characteristics were different between two cultivars, and hence the sampling and analysis were conducted separately. In the positive ion mode, the platform detected 3316 DRMs, of which 1706 and 1610 compounds were up- and downregulated in “JNYB”; in the negative ion mode, 1548 upregulated and 1490 downregulated metabolites were detected. Significantly fewer compounds were identified in “DHBC”: 92 upregulated and 110 downregulated metabolites were found in the positive ion mode; similarly, 65 and 112 up- and downregulated compounds were identified in the negative ion mode (**Figure 3A**). The information of DRMs and their fold change (ratio), besides details on up- and downregulated metabolites, is provided in **Supplementary Table S2**. The hierarchical clustering analysis further classified these metabolites into two major groups based on their regulation pattern in both clubroot-susceptible and clubroot- resistant cultivars; the mock and diseased samples were clearly separated (**Supplementary Figure S3**). The combined analysis was conducted. A total of 3254 upregulated compounds were detected in clubroot-diseased “JNYB” roots compared with healthy counterparts, whereas 157 upregulated were detected in “DHBC” (**Figure 3B**). Among downregulated metabolites, 3104 (3100 + 4) compounds were differentially detected in diseased “JNYB” samples, and 226 (222 + 4) were detected in “DHBC” diseased root samples (**Figure 3B**). 15 significantly DRMs were detected in both in “JNYB” and “DHBC”, among them M617T251, M287T355 and M229T43 were downregulated in both cultivras, but no definite names were given; further, 12 metabolites were found to be up or down regulated in each cultivar; half of them were lipids and lipid-like molecules (**Supplementary Table S3**).

PCA was performed to reduce the dimensionality of the data and visualize the relationship among samples of two cultivars. In “JNYB”, the first principal component (PC1) explained 45.43% of the total variation, while the second principal component (PC2) explained 6.02% variation across the data set. The score plot between the PC1 and PC2 showed clear separation by PC1 between *P. brassicae*–infected roots and mock in “JNYB” (**Figure 3C**). In “DHBC”, the PC1 explained 51.38% of the total variation, and PC2 explained 12.75% variation across the data set. The score plot between the PC1 and PC2 showed less separation by PC1 between *P. brassicae*–infected “DHBC” roots and mock (**Figure 3C**). The PLS-DA was performed as well. *P. brassicae* infection treatment explained 58.11% of PC1 and 4.38% of PC2 in “JNYB” and 15.16% of PC1 and 12.52% of PC2 in “DHBC”, respectively. The score plot between the PC1 and PC2 showed clear separation by PC1 between *P. brassicae*–infected roots and mock in both cultivars, but a higher level of separation was found on “JNYB” (**Supplementary Figure S4**). According to the *P* value and VIP score, the top 50 clubroot-responsive metabolites in “JNYB” and “DHBC” and their changing patterns are presented in **Figure 3D**. Very limited compounds were noted with a definite name: in the “JNYB”, 7-hydroxy-4-(methyl)coumarin, sinapic acid, and sinapoyl malate were upregulated in diseased samples, while honyucitrin, IYSOpe 20:4, lysoPC 20:4, 1-arachidonoylglycerol, 12R-hydroxy-5Z,8Z,10E,14Z-eicosateraenoic acid, retinyl ester, and *cis*-8,11,14,17-eicosatetraenoic acid were downregulated; In the “DHBC”, clubroot led to apiosylglucosyl 4-hydroxybenzoate upregulation, while LysoPE 18:20, LysoPC 16:0, LysoPE 16:0, LysoPC 18:3, Ginsenoside-Rg1, almitoyl-hydroxy-sn-glycerol-3-phosphoethanolamine, LysoPC 18:1, LysoPE 18:1, and LysoPE 18:3 were downregulated (**Figure 3D**).

Five and nine KEGG pathways were significantly regulated by *P. brassicae* infection in the cortex splitting stage of two cultivars, respectively (**Table 2**). In “JNYB”, anthocyanin biosynthesis (11 DRMs of 66 background metabolites), glucosinolate biosynthesis (11 DRMs of 75 background metabolites), carbapenem biosynthesis (5 DRMs of 32 background metabolites), selenocompound metabolism (4 DRMs of 27 background metabolites), and flavone and flavonol biosynthesis (6 DRMs of 49 background metabolites) were found to be significantly regulated; in “DHBC”, stilbenoid, diarylheptanoid, and gingerol biosynthesis, cutin, suberine, and wax biosynthesis, alanine, aspartate, and glutamate metabolism, thiamine metabolism, beta-alanine metabolism, alpha-linolenic acid metabolism, histidine metabolism, isoflavonoid biosynthesis, and tropane, piperidine, and pyridine alkaloid biosynthesis were significantly regulated; a single DRM was identified in each pathway (**Table 2**). Detailed information of each pathway see **Supplementary Table S4**. Glucosinolate and carbapenem were selected from significant DRMs or pathways from “JNYB” respectively, ginsenoside was selected form “DHBC”. The gingerols were detected in diseased “DHBC”, although no significant differences were detected. Considering the shared functions between ginsenoside and gingerol in clinic use, gingerol was selected as well. These four kinds of metabolites were used in the following experiments, details of carbapenem biosynthesis see **Supplementary Table S4**, the information of secondary metabolites of glucosinolate and ginsenoside see **Supplementary Table S5**.

**Table 2:** KEGG pathways of “JNYB” and “DHBC” significantly regulated in response to clubroot.

KEGG pathway (ID)	No. of DRM	No. of background metabolites	No. of features	*P* value
“JNYB”				
Anthocyanin biosynthesis (00942)	11	66	15	0.001321
Glucosinolate biosynthesis (00966)	11	75	16	0.004056
Carbapenem biosynthesis (00332)	5	32	8	0.018566
Selenocompound metabolism (00450)	4	27	4	0.032399
Flavone and flavonol biosynthesis(00944)	6	49	9	0.044949
“DHBC”				
Stilbenoid, diarylheptanoid, and gingerol biosynthesis (00945)	1	25	1	0.005128
Cutin, suberine, and wax biosynthesis (00073)	1	27	1	0.005968
Alanine, aspartate, and glutamate metabolism (00250)	1	28	1	0.006411
Thiamine metabolism (00730)	1	31	1	0.007826
Beta-alanine metabolism (00410)	1	32	1	0.008326
Alpha-linolenic acid metabolism (00592)	1	42	1	0.014086
Histidine metabolism (00340)	1	47	1	0.017459
Isoflavonoid biosynthesis (00943)	1	63	1	0.030272
Tropane, piperidine, and pyridine alkaloid biosynthesis (00960)	1	68	1	0.03486

### 3.3 Effects of metabolites on induced clubroot resistance and growth of radish “JNYB”

#### 3.3.1 Effects of metabolites on induced clubroot resistance

**Figure 4:**
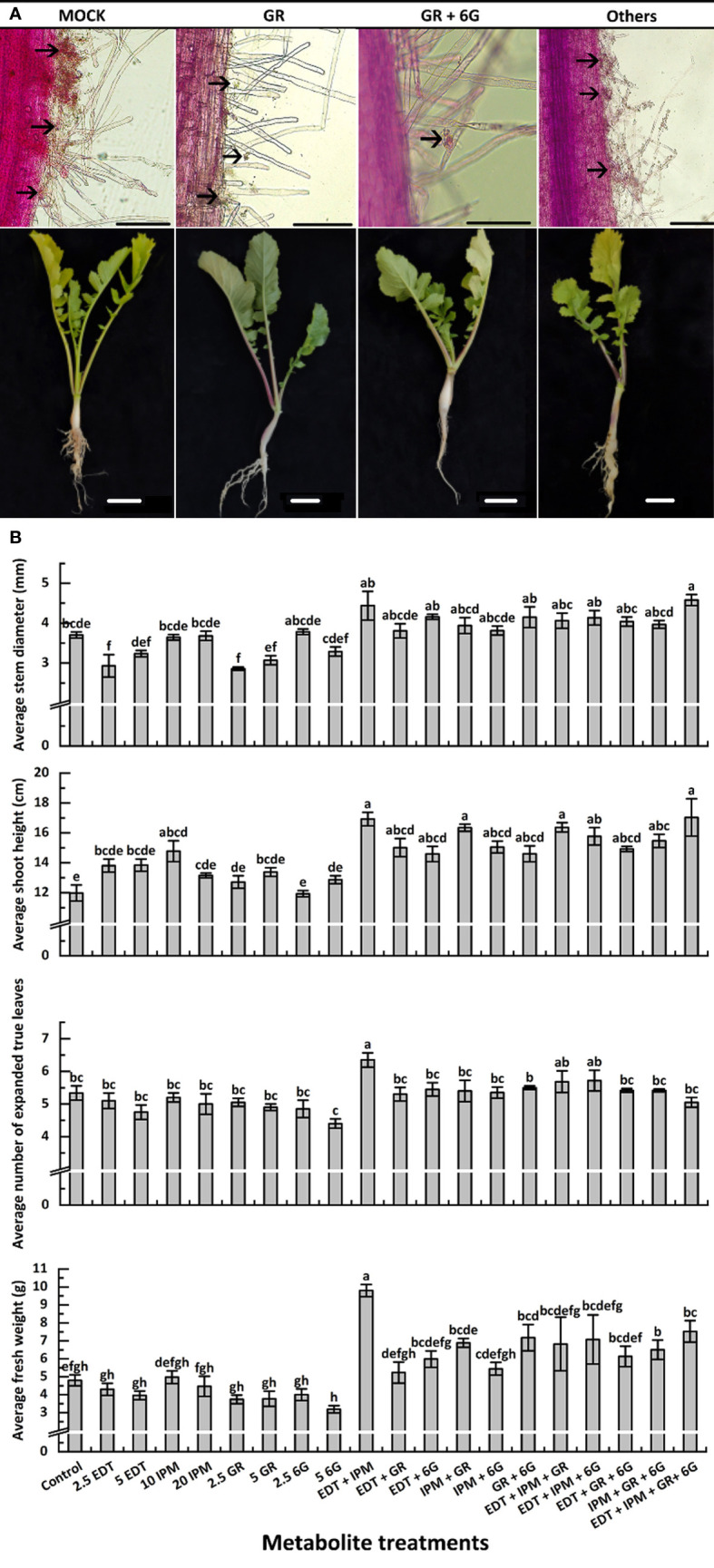
Effects of metabolites on root club formation of diseased “JNYB” seedlings **(A)** and growth of infected seedlings **(B)**. “other” in **(A)** refers to other treatments; for details, see **Supplementary Figures 5–8**. The first line indicates the growth of *P. brassicae* within the root hair of radish 24 h after inoculation with resting spores; the second line exhibits the clubroot disease incidence in radish tap root in the cortex splitting stage. Black arrows in **(A)** indicate spores. Scale bar in first and second lines equals to 100 μm and 1 cm, respectively. Data in **(B)** are presented by mean value ± SE. A total of 20 randomly selected samples were treated and measured in each replication. Three replications were conducted. Letters indicate significant differences. Significant differences were analyzed on *P* < 0.05.

The seedlings were treated with metabolites solution of different concentrations and their mixture (**Table 3**), and mocks were treated with an equal volume of their solvent, to explore whether DRMs, such as EDT, IPM, GR, and 6G, increased “JNYB” resistance to *P. brassicae*. The disease incidence and index of mock were up to 77.1% and 32.0, respectively (**Table 3**). In the single metabolism treatments, except for EDT, the seedlings treated with IPM, GR, and 6G exhibited limited root galls, the disease incidence decreased up to 36.8%, and the disease index ranged from 12.2 to 21.6 in all treatments. Further, 5 mg/L GR treatment resulted in the least disease symptoms (**Table 3**). On testing with hydroponic culture, a significantly less number of spores enriched in root hair were identified compared with mock. In substrate cultivation, swelled lateral roots were observed, but very few symptoms were detected on the taproot (**Figure 4A**). Compared with the mock, dual metabolite processing significantly reduced the symptom of swelled roots; disease incidence of 10.0%–50.2% and disease index of 3.3–19.4 were detected. Very few galls were found in the roots of plants treated with GR + 6G. Only 10.0% of the samples were found to be diseased, and the disease index was 3.3. The enrichment of spores in root hair in hydroponic culture was rarely seen, and a majority of samples had healthy taproots (**Table 3** and **Figure 4A**). The most serious club symptom was observed on treatment with EDT + 6G (**Table 3**). In the triple treatments, the combinations resulted in 28.2%–47.4% of disease incidence and 9.1−15.6 of disease index, which were higher than those of some of the samples treated with two metabolites, such as GR + 6G and IPM + GR. The highest reduction of root clubs was observed on treatment with IPM + GR + 6G. The least effect was found on treatment with EDT + IPM + 6G. EDT + IPM + GR + 6G led to 25.5% of disease incidence and 8.3 of disease index (**Table 3**). Generally speaking, IPM, GR, and 6G could inhibit root club formation under the present test condition, 5 mg/L GR worked the finest among single metabolite treatments, and the combination of GR and 6G was the most efficient in inducing the radish resistance in response to clubroot disease in all treatments. A significant decrease in disease symptoms was found on IPM treatments; however, IPM did not promote the anti-clubroot efficiency in any of the combinations. EDT use alone did not help seedlings to develop resistance to *P. brassicae*; when combined with other metabolites, it could even offset the promoting effects (**Table 3**). The effects of metabolites on spore enrichment, root galls inhibition under hydroponic culture and substrate culture are shown in **Supplementary Figures S5-8**.

**Table 3:** Effects of metabolite treatments on the occurrence of clubroot on sensitive radish cultivar “JNYB”.

Treatments	Disease incidence	Disease index	Spore titer (RT-qPCR)	Treatments	Disease incidence	Disease index	Spore titer (RT-qPCR)
ddH2O	77.1 ± 0.2 a	32.0 ± 2.8 a	0.950 ± 0.027 b	EDT + GR	45.3 ± 0.0 def	8.3 ± 1.7 fg	0.244 ± 0.007 c
2.5 EDT	70.7 ± 0.3 ab	30.0 ± 2.1 a	1.430 ± 0.033 a	EDT + 6G	50.2 ± 0.1 bcd	19.4 ± 2.9 bc	0.003 ± 0.000 f
5 EDT	75.2 ± 0.1 a	30.0 ± 3.5 a	0.022 ± 0.003 ef	IPM + GR	25.5 ± 0.1 fg	9.5 ± 1.7 efg	0.090 ± 0.082 def
10 IPM	50.3 ± 0.0 bcd	17. 8 ± 0.0 bcd	0.026 ± 0.006 ef	IPM + 6G	50.1 ± 0.2 bcd	17.8 ± 2.7 bcd	0.053 ± 0.021 def
20 IPM	50.2 ± 0.1 bcd	21.1 ± 3.3 b	0.025 ± 0.016 ef	GR + 6G	10.0 ± 0.1 fg	3.3 ± 1.9 g	0.011 ± 0.002 f
2.5 GR	50.3 ± 0.1 bcd	17.2 ± 1.7 bcde	0.008 ± 0.003 f	EDT + IPM + GR	37.7 ± 0.0 def	9.1 ± 0.5 fg	0.107 ± 0.011 de
5 GR	40.3 ± 0.1 def	12.2 ± 1.9 cdef	0.039 ± 0.013 def	EDT + IPM + 6G	47.4 ± 0.0 cde	15.6 ± 2.6 bcdef	0.013 ± 0.002 f
2.5 6G	50.2 ± 0.1 bcd	18.9 ± 3.2 bc	0.057 ± 0.041 def	EDT + GR + 6G	35.2 ± 0.1 def	10.7 ± 3.6 defg	0.010 ± 0.001 f
5 6G	50.4 ± 0.0 bcd	21.6 ± 2.1 b	0.125 ± 0.057 d	IPM + GR + 6G	28.2 ± 0.1 fg	10.2 ± 2.7 defg	0.011 ± 0.001 f
EDT + IPM	40.7 ± 0.0 def	9.5 ± 1.7 efg	0.014 ± 0.003 f	EDT + IPM + GR + 6G	25.5 ± 0.1 fg	8.3 ± 1.7 fg	0.035 ± 0.020 ef

A total of 20 randomly selected samples were treated and measured in each replication. Three replications were conducted. Data are presented by mean value ± SE. Letters indicate significant differences. Significant difference were analyzed on P < 0.05.

RT-qPCR was conducted to further verify the effects of exogenous metabolite treatments. The results were consistent with the disease incidence and disease index survey. Further, 2.5 mg/L EDT (1.430) and mock (0.950) resulted in the highest spore titer, while the lowest (0.008) was detected on 5 mg/L GR–treated samples in single metabolite treatments. The titer of *P. brassicae* in GR + 6G treatment (0.011) was significantly lower than that of mock. Generally speaking, except for EDT, the single metabolite treatments and each combination significantly inhibited the *P. brassicae* proliferation (**Table 3**).

#### 3.3.2 Effects of metabolites on the growth of infected radish “JNYB”

The morphological parameters varied among different metabolite treatments. Generally, each metabolite used alone slightly inhibited or did not affect the growth or morphology, while the synergistic promotion effects were witnessed. Dual or triple metabolite treatments significantly improved seedling growth. Quadruple processing by all metabolites significantly enhanced seedling growth in terms of indexes such as stem diameter, shoot height, and FW, but not in terms of the number of leaves (**Figure 4B**). In detail, EDT and IPM did not significantly affect radish growth, while their dual combination resulted in the highest vitality of seedlings among all treatments. Moreover, EDT and IPM combination with other metabolites exhibited growth promotion (**Figure 4B**). The GR, which was the most efficient in reducing disease symptoms displayed negative effects on stem expansion and dry matter accumulation compared with mock. It slightly decreased leaf number, but this effect was not significant; the promoting effects were only detected on shoot height (**Figure 4B**). Also, 5 mg/L 6G inhibited the seedling growth; however, the combination of two compounds significantly promoted all growth parameters, 4.18 mm of stem diameter, 14.5 cm of shoot length, 5.6 of leaves, and 7.2 g of FW were measured, while 3.7 mm of stem diameter, 11.9 cm of shoot length, 5.3 of leaf number, and 4.7 g of FW were detected on mock plants. In summary, the application of 5 mg/L GR + 5 mg/L 6G was the best choice for inducing clubroot disease resistance, since it inhibited symptom development and promoted seedling growth at the same time (**Figure 4B**).

### 3.4 Effects of *P. brassicae* infection on the carbapenem pathway and ginsenoside metabolism

**Figure 5:**
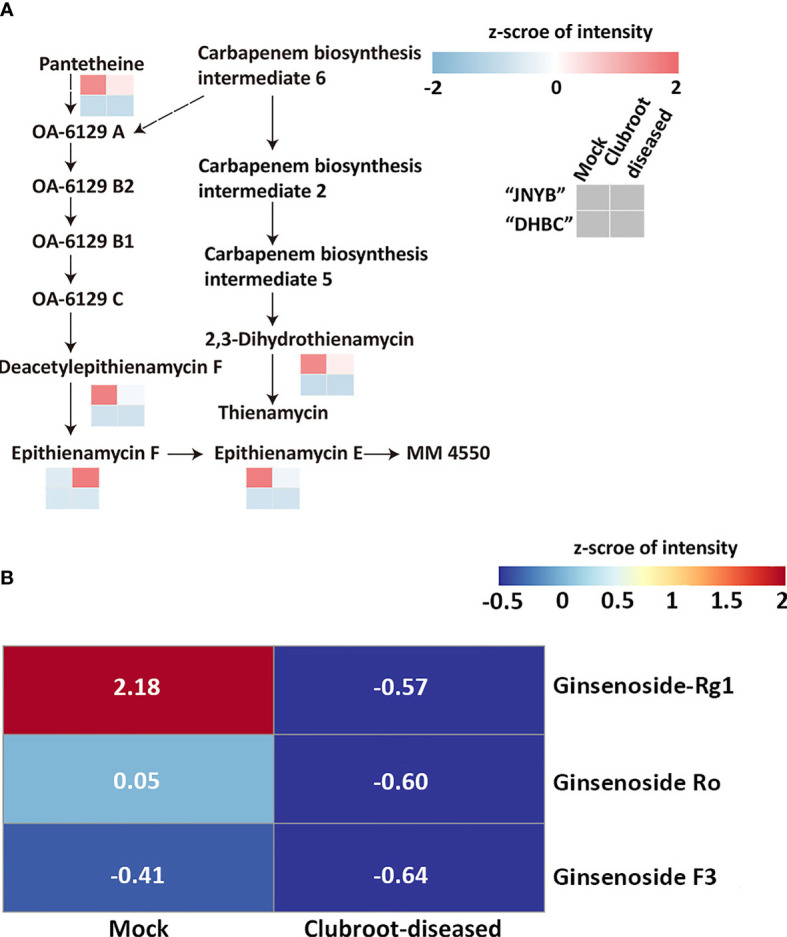
Analysis of carbapenem pathway **(A)** and ginsenoside metabolism **(B)** affected by *P. brassicae* infection. Data were normalization processed.

Based on the results of anti-clubroot assessment of the aforementioned metabolites, DRMs were analyzed in the carbapenem biosynthesis pathway (00332). Meanwhile, no pathway information of ginsenoside biosynthesis was found, and thus the metabolite changes were analyzed. Although 6-gingerol and 8- gingerol were detected or calculated in “DHBC” and “JNYB” (data not shown), significant changes and related pathway information were not seen. In “JNYB”, five compounds in total were differentially regulated in carbapenem biosynthesis, which were pantetheine, 2,3-dihydro-thienamycin (substrate for thienamycin biosynthesis), epithienamycin F, epithienamycin E, and MM4550 (C20815). Four of them were downregulated by *P. brassicae* infection, and only epithienamycin E was upregulated. The biosynthesis of these compounds was rarely detected in “DHBC” (**Figure 5A**). Ginsenoside Rg1, Ro, and F3 were detected in “DHBC,” but not in “JNYB” (**Figure 5B**). The synthesis of ginsenoside in mock was unstable, big difference of value of ginsenoside compounds of 6 samples were detected (**Supplementary Table S6**), we consider this may cause by its hybrid characteristic, however, according to the average value, *P. brassicae* infection significantly decreased the content of ginsenosides.

## 4 Discussion

Considering the complexity of the interaction between *P. brassicae* and host crops, cultivating resistant varieties are thought to be the most efficient strategy for controlling clubroot disease. Therefore, this study was performed to understand the complex mechanisms underlying the *P. brassicae* response in susceptible and resistant radish cultivars, the biochemical events involved in clubroot resistance conferred by the metabonomes were profiled and compared by the LC-MS/MS of radish cultivars with different resistance to *P. brassicae*. We applied the acquired knowledge, and search for biocontrol metabolites for clubroot disease.

The researches focusing on clubroot pathogenesis have a long history (Ayers, 1944; Kageyama and Asano, 2009); however, its pathogenesis in radish is not clear yet. Considering that the morphology and developmental characteristics significantly vary between radish and other plants, the trend of spore infection and symptom appearance that has been reported previously may not be referential. Therefore, we first studied how *P. brassicae* infected root hairs, how root clubs arose, and the differences between susceptible and resistant cultivars. Previously, the dual culture of root and resting spores was used to illustrate the primary infection by resting spores on host root hairs (Asano et al., 2000; Sharma et al., 2011). This method allowed successful serial observation of root hair infection in a sample (Asano et al., 2000). However, root hairs without shoots may differ physiologically and biochemically from those of intact plants (Kageyama and Asano, 2009). Thus, a hydroponic culture with intact seedings and resting spores was conducted to simulate the actual infection state. As expected, the developmental stages observed in radish in the present study were similar to those reported previously in other cruciferous hosts. The resting spores formed primary zoospores 24 h after inoculation, and the formation of multinucleate zoosporangial plasmodium and the zoospore release was thereafter identified by 72 h after inoculation (**Figure 2**). Root hair infection was observed from 18 h to 2 days after inoculation on *B. rapa* (Ingram and Tommerup, 1972; Sharma et al., 2011), which was consistent with our results. Moreover, the pathogen proliferating in a short cycle around root hairs was identified as reported earlier, which enhanced the infection of cortical tissues in the root hair infection stage (Kageyama and Asano, 2009). However, a difference was observed in the time lag from spore inoculation to the formation of multinucleate zoosporangial plasmodium. Its formation was reported 6–8 days after inoculation (Naiki and Dixon, 1987; Asano et al., 2000; Donald and Porter, 2004), while 3 days (72 h) were merely needed for multinucleate zoosporangial plasmodium development in radish root hairs; this might be due to the differences in host genotype and incubation environment compared with previous studies. Interestingly, no significant differences were observed in the time lag for spore infection, proliferation and clubroot appearance between susceptible and resistant cultivars (**Figure 2** and **Table 1**). Studies confirmed that *P. brassicae* root hair infection was observed in nonhost and host plants (Kageyama and Asano, 2009). We further verified that the infection state was similar in both susceptible and resistant cultivars. However, whether the secondary zoospores from resistant cultivar roots had similar virulence in attacking susceptible cultivars was not clear. The root clubs were primarily generated in the cortex splitting stage of radish and then rapid formatted in the post-cortex splitting stage (**Figure 2** and **Table 1**). According to the life cycle of *P. brassicae*, cortical infection was followed by spore germination and root hair infection. Inside infected cortex cells, the pathogen developed into secondary plasmodia, which proliferated and were associated with cellular hypertrophy; thereafter, root galls were generated (Kageyama and Asano, 2009). We preliminarily hypothesized that the cells rapidly proliferated in the cortex splitting stage. The infected cells might also divide rapidly. Moreover, diseased cells might stimulate the level of phytohormones such as cytokinins (Laila et al., 2020) in the swelling cortex, thus leading to the malformed root and gall formation.

Metabonomic studies offered important information on the molecular mechanism of *P. brassicae* and host interaction, which mutually supported the physiological and “omic” studies. Pedras et al. (2008) conducted a metabonomic study using clubroot-diseased *B. napus* by the HPLC-NMR method. Compared with the mock, higher concentrations of phytoalexins were measured in the fifth and sixth weeks after inoculation, and phytoanticipins were significantly upregulated in the third or fourth week after inoculation. The JA and phytoalexins, including flavonoid and indole-containing compounds, were found to participate in the defensive response induced by clubroot-resistant gene *Rcr 1* in *B. rapa* by utral high-pressure liquid chromatography-MS/MS (UHPLC-MS/MS) analysis (Song et al., 2015). The JA signaling pathway (Prerostova et al., 2018; Fu et al., 2019) and flavonoid biosynthesis (Irani et al., 2019) were found to be involved in the biological process of clubroot development by transcriptomic analysis. In addition, phytoalexins and phytoanticipins were involved in the plant defense response. These results further confirmed the practicability of metabonomic analysis in the studies of host–pathogen interaction.

A metabonomic study was conducted first on radish in the present study. A significantly larger number of DRMs were detected in susceptible cultivar “JNYB” than in “DHBC” (**Figures 3A, B**). Moreover, in the initial stage of clubroot onset, the metabolite profiles of healthy and diseased “JNYB” roots were discrete compared with those in “DHBC” (**Figure 3C**). These results indicated a more stable metabolic level of resistance cultivar in response to *P. brassicae* infection. Interestingly, lipids and lipid-like molecules were both differentially regulated between both cultivars, while they were upregulated in “JNYB” and downregulated in “DHBC”. Whether these molecules can be used as indicators of clubroot resistance in susceptible or nonsusceptible cultivars needs further exploration (**Supplementary Table S3**). Based on the *P* value and VIP score, significantly regulated pathways and metabolites were selected. Five pathways were significantly regulated (**Table 2**), in which the metabolism of glucosinolate (Zhang et al., 2016; Li et al., 2019; Zamani-Noor et al., 2021), selenocompound (Wei et al., 2016), and flavonoid (Song et al., 2014; Irani et al., 2019) in response to *P. brassicae* infection was reported; however, anthocyanin and carbapenem biosyntheses were not mentioned. The significantly regulated KEGG pathways in diseased “DHBC” roots were seldom reported previously in other diseased crops, indicating that a relatively unique molecular response might take place in this genotype (**Table 2**).

In cruciferous crops, glucosinolates are often related to not only flavor and quality but also *P. brassicae* infection and clubroot resistance (Ludwig-Müller et al., 1997; Ludwig-Muller et al., 1999; Zamani-Noor et al., 2021). The upregulation of glucosinolate biosynthesis was frequently found in diseased roots (Zhao et al., 2017), leading to a pungent smell. Some glucosinolate metabolites have been found to be toxic to pests such as insects and some fungal pathogens and play important roles in the plant’s defense response (Fahey et al., 2001; Agerbirk and Olsen, 2012). However, in the present study, EDT, which belonged to glucosinolates, failed to promote the resistance of radish to *P. brassicae* infection. Moreover, this metabolite might slightly offset the positive effects induced by GR + 6G (**Table 3**). Thus, EDT might not be applied as an anti-*P. brassicae* inducer. The function of other compounds needs further verification. The carbapenems, including IPM, meropenem, doripenem, and other new members, are β-lactam antimicrobial agents with an exceptionally broad spectrum of activity toward many Gram-positive, Gram-negative, and anaerobic bacteria (Nicolau, 2008). They are often used against serious infections (Norrby, 2018). However, the extensive clinical application of these compounds led to the generation of carbapenem-resistant microbes and has caused serious public health concerns (Bricch et al., 2020). The pathway of carbapenem biosynthesis (00332) was significantly regulated by *P. brassicae* infection in “JNYB”. As naturally produced metabolites, carbapenems can be synthesized and isolated from phytopathogens such as *Erwinia carotovora* (Byers et al., 2002) and *Pectobacterium carotovorum* subsp. *carotovorum* ATCC 39048 (Monson et al., 2018), while their biosynthesis has not been reported in *P. brassicae*. The above results indicate a possible effect between sensitive radish cultivar and spores in carbapenems biosynthesis and metabolize. Compared with mock, IPM significantly inhibited the incidence and severity of root clubs *via* exogenous application alone or in combination with other metabolites (**Table 3**); this effect was less than that of ginsenoside treatment. The ginsenosides have been widely regarded as natural products with a broad spectrum of clinical use (Xu et al., 2021). Robust evidence has been provided for the remarkable role of ginsenosides in cardiovascular diseases, nervous system diseases, metabolic diseases, and certain types of cancer (Zhou et al., 2019; Wang et al., 2020) and antimicrobial response (Xue et al., 2020). The anti-cancer activity of ginsenosides has been confirmed in breast (Kim and Kim, 2015; Lee et al., 2018), colonic (Chen et al., 2016b), gastric (Mao et al., 2014; Qian et al., 2016), glioma (Li et al., 2018; Gu et al., 2019), hepatic (Chen et al., 2017; Wang et al., 2018), leukemia (Zhuang et al., 2018), prostate (Chen et al., 2016b; Peng et al., 2019), pulmonary (Ge et al., 2017; Wang et al., 2018), and ovarian (Li et al., 2015; Zhou et al., 2018) cancer cells. These compouds are mainly synthezised in herb medicines such as *Panax ginseng* (Jung et al., 2014) and *Panax notoginseng* (Li et al., 2022).While Strain PDA-2, which belongs to the genus Agrobacterium and can be isolated from ginseng roots showed high capacity in producing rare ginsenosides (Yan et al., 2019). At present, the active synthesis of ginsenosides in radish has not been reported, but the synthesis related enzyme UDP glucuronosyltransferase super family (Li et al., 2022) can be found in radish genome (data not shown). The cross-talk of gingerol and its anti-cancerous potential has long been investigated in breast (Mohammed, 2021), lung (Geng et al., 2016), and colorectum (Yusof et al., 2021) cancers. Moreover, the antimicrobial activity of this compound was also reported (Chiaramonte et al., 2021). Using transcriptomic analysis, Bi et al. (2018) detected 171 proto-oncogenes from the genome of *P. brassicae* strain ZJ-1, which were implicated in cancer-related pathways. Three predicted proto-oncogenes showed homology to the human proto-oncogenes *Raf* and *MYB*. They were specifically activated during the plasmodial growth in host cortical cells, indicating that the proliferation of *P. brassicae* spores shared similarities with cancer cells. In the present study, 5 mg/L ginsenoside resulted in the lowest disease incidence of radish seedlings during signaling metabolite treatments, while the addition of 5 mg/L 6-gingerol further strengthed the anti-*P. brassicae* ability. Considering the anti-cancer functions of ginsenoside and gingerol, whether the clubroot inhibition effects of these two metabolites are related to the regulation of *P. brassicae* proto-oncogenes deserves further exploration.

The effects of metabolites on the seedling growth of radish “JNYB” were assessed. In general, the highest growth promotion was found after IPM + EDT dual treatment, followed by quadruple compound treatment (**Figure 4B**). Interestingly, 5 mg/L GR or 6G used alone decreased the growth indexes of stem diameter, number of leaves, and biomass accumulation, while the combination of these two metabolites significantly enhanced the growth indexes (**Figure 4B**). This result was verified twice. Further studies should be conducted to reveal the mechanism underlying this effect. Comprehensively considering the disease inhibition effect of these four metabolites and their impact on growth, the dual application of 5 mg/L GR and 5 mg/L 6G was considered to be the most effective; however, more carbapenem members should be tested.

The *P. brassicae* infection significantly decreased the composition of 2,3-dihydro-thienamycin, which was responsible for thienamycin synthesis, and the epithienamycin F in carbapenem biosynthesis, while exogenous application of IPM, which was considered to be the first thienamycin antibiotic (Geddes and Stille, 1985), reversed the root club development. The present result might indicate a possibility that during *P. brassicae* infection, the spores might turn off the carbapenem biosynthesis to reduce the pressure for their invasion or proliferation, since both thienamycin and epithienamycin were antibiotics. A similar situation was identified in ginsenoside biosynthesis (**Figure 5**). Considering its anti-cancer characteristic, *P. brassicae* may accelerate proliferation by highly expressing proto-oncogenes through inhibiting ginsenoside levels in the host. These findings might offer new insights in revealing the interaction mechanisms between radish and *P. brassicae*.

The current metabonomic studies and preliminary metabolite verification should be expanded to investigate the efficacy of carbapenem, ginsenoside, and gingerol as *in vivo* anti-*P. brassicae* agents. It has been previously shown that these compounds are beneficial as antimicrobial and anti-cancer agents. Also, the proliferation of *P. brassicae* shares similarity with cancer cells. These results provided an solid foundation for identifying critical radish-original anti-*P. brassicae* agents and would facilitate further dissecting metabolic mechanisms underlying clubroot defense and resistance in radish.

## Data availability statement

The data presented in the study are deposited in the MetaboLights (https://www.ebi.ac.uk/metabolights) repository, accession number MTBLS6347 and MTBLS6348.

## Author contributions

JLi: writing original draft preparation. JLi and TH:writing – review and editing. JLi, TH and JLu: methodology. XX and WZ: resources. JLi, TH and JLu: data analysis. All authors contributed to the article and approved the submitted version.

## References

[B1] Agerbirk N. Olsen C. E. (2012). Glucosinolate structures in evolution. Phytochemistry 77, 16–45. doi: 10.1016/j.phytochem.2012.02.005 22405332

[B2] Akaba M. Kaneko Y. Hatakeyama K. Ishida M. Bang S. W. Matsuzawa Y. (2009). Identification and evaluation of clubroot resistance of radish chromosome using a *Brassica napus*-*Raphanus sativus* monosomic addition line. Breed Sci. 59, 203–206. doi: 10.1270/jsbbs.59.203

[B3] Arif S. Liaquat F. Yang S. Shah I. H. Zhao L. Xiong X.. (2021). Exogenous inoculation of endophytic bacterium *Bacillus cereus* suppresses clubroot (*Plasmodiophora brassicae*) occurrence in pak choi (*Brassica campestris* sp. *chinensis* l.). Planta 253, 25. doi: 10.1007/s00425-020-03546-4 33404767

[B4] Asano T. Kageyama K. Hyakumachi M. (2000). Germination of surface-disinfected resting spores of *Plasmodiophora brassicae* and their root hair infection in turnip hairy roots. Mycoscience 41, 49–54. doi: 10.1007/BF02464385

[B5] Ayers G. W. (1944). Studies on the life history of the club root organism, *Plasmodiophora brassicae* . Can. J. Res. 22, 143–149. doi: 10.1139/cjr44c-012

[B6] Barrett D. P. Fowler S. V. Subbaraj A. K. Groenteman R. Clavijo-McCormick A. (2021). Metabolomic analysis of host plant biochemistry could improve the effectiveness and safety of classical weed biocontrol. Biol. Control. 160, 104663. doi: 10.1016/j.biocontrol.2021.104663

[B7] Bi K. Chen T. He Z. Gao Z. Zhao Y. Fu Y.. (2018). Proto-oncogenes in a eukaryotic unicellular organism play essential roles in plasmodial growth in host cells. BMC Genomics 19, 881. doi: 10.1186/s12864-018-5307-4 30522435 PMC6282348

[B8] Bricch M. Ast A. Pascale A. Andren F. Pavan A. (2020). Implementation of preventive actions to control carbapenem-resistant enterobacteriaceae and mdr gram negatives at a neurological hospital. J. Microbiol. Infect. Dis. 10, 1–9. doi: 10.5799/jmid.700466

[B9] Byers J. T. Lucas C. Salmond G. Welch M. (2002). Nonenzymatic turnover of an *Erwinia carotovora* quorum-sensing signaling molecule. J. Bacteriology 184 (4), 1163–1171. doi: 10.1128/jb.184.4.1163-1171.2002 PMC134803 11807077

[B10] Cao T. S. Srivastava S. Rahman M. H. Kav N. N. V. Hotte N. Deyholos M. K.. (2008). Proteome-level changes in the roots of *Brassica napus* as a result of *Plasmodiophora brassicae* infection. Plant Sci. J. 174, 97–115. doi: 10.1016/j.plantsci.2007.10.002

[B11] Chen F. B. Xing C. Y. Huo S. P. Cao C. L. Yao Q. L. Fang P. (2016a). Red pigment content and expression of genes related to anthocyanin biosynthesis in radishes (*Raphanus sativus* l.) with different colored flesh. J. Agric. Sci. 8, 126. doi: 10.5539/jas.v8n8p126

[B12] Chen F. Sun Y. Zheng S. L. Qin Y. Julian M. D. Hu J. N.. (2017). Antitumor and immunomodulatory effects of ginsenoside Rh2 and its octyl ester derivative in H22 tumor-bearing mice. J. Funct. Foods 32, 382–390. doi: 10.1016/j.jff.2017.03.013

[B13] Chen L. Meng Y. Sun Q. Zhang Z. Y. Guo X. Q. Sheng X. T.. (2016b). Ginsenoside compound K sensitizes human colon cancer cells to TRAIL-induced apoptosis *via* autophagy-dependent and independent DR5 upregulation. Cell Death Dis. 7, e2334. doi: 10.1038/cddis.2016.234 27512955 PMC5108320

[B14] Chiaramonte M. Bonaventura R. Costa C. Zito F. Russo R. (2021). 6-gingerol dose-dependent toxicity, its role against lipopolysaccharide insult in sea urchin (*Paracentrotus lividus* lamarck), and antimicrobial activity. Food Biosci. 39, 100833. doi: 10.1016/j.fbio.2020.100833

[B15] Dixon G. R. (2009). The occurrence and economic impact of *Plasmodiophora brassicae* and clubroot disease. J. Plant Growth Regul. 28, 194–202. doi: 10.1103/PhysRevB.56.12529

[B16] Donald E. C. Porter I. J. (2004). A sand-solution culture technique used to observe the effect of calcium and pH on root hair and cortical stages of infection by *Plasmodiophora brassicae* . Australas. Plant Pathol. 33, 585–589. doi: 10.1071/AP04068

[B17] Donald C. Porter I. J. (2009). Integrated control of clubroot. J. Plant Growth Regul. 28, 289–303. doi: 10.1007/s00344-009-9094-7

[B18] Fahey J. W. Zalcmann A. T. Talalay P. (2001). The chemical diversity and distribution of glucosinolates and isothiocyanates among plants. Phytochemistry 56, 5–51. doi: 10.1002/chin.200110286 11198818

[B19] Fu P. Y. Piao Y. L. Zhan Z. X. Zhao Y. Z. Pang W. X. Li X. N.. (2019). Transcriptome arofile of *Brassica rapa* l. reveals the involvement of jasmonic acid, ethylene, and brassinosteroid signaling pathways in clubroot resistance. Agron. J. 9, 589. doi: 10.3390/agronomy9100589

[B20] Galindo-González L. Manolii V. Hwang S. F. Strelkov S. E. (2020). Response of Brassica napus to Plasmodiophora brassicae involves salicylic acid-mediated immunity: An RNA-Seq-based study. Front. Plant Sci. 11, 1025. doi: 10.3389/fpls.2020.01025 32754180 PMC7367028

[B21] Gazengel K. Aigu Y. Lariagon C. Humeau M. Daval S. (2021). Nitrogen supply and host-plant genotype modulate the transcriptomic profile of *Plasmodiophora brassicae* . Front. Microbiol. 12. doi: 10.3389/fmicb.2021.701067 PMC8298192 34305867

[B22] Geddes A. M. Stille W. (1985). Imipenem: the first thienamycin antibiotic. Rev. Infect. Dis. Suppl 3, S353–S356. doi: 10.1093/clinids/7.supplement_3.s353 3863216

[B23] Geng S. G. Zheng Y. Q. Meng M. J. Guo Z. Z. (2016). Gingerol reverses the cancer-promoting effect of capsaicin by increased TRPV1 level in a urethane-induced lung carcinogenic model. J. Agric. Food Chem. 64, 31. doi: 10.1021/acs.jafc.6b02480 27436516

[B24] Ge G. Q. Yan Y. Cai H. (2017). Ginsenoside Rh2 inhibited proliferation by inducing ROS mediated ER stress dependent apoptosis in lung cancer cells. Biol. Pharm. Bull. 40, 2117–2124. doi: 10.1248/bpb.b17-00463 28966297

[B25] Gu B. Wang J. Song Y. Wang Q. Wu Q. (2019). The inhibitory effects of ginsenoside Rd on the human glioma u251 cells and its underlying mechanisms. J. Cell. Biochem. 120, 4444–4450. doi: 10.1002/jcb.27732 30260020

[B26] Hartley S. E. Gange A. C. (2009). Impacts of plant symbiotic fungi on insect herbivores: mutualism in a multitrophic context. Annu. Rev. Entomol. 54, 323–342. doi: 10.1146/annurev.ento.54.110807.090614 19067635

[B27] Hirani A. H. Li G. (2015). Understanding the genetics of clubroot resistance for effectively controlling this disease in Brassica Species. In Plants for the Future edited by El-Shemy H.(London: IntechOpen)2015, 10.5772/60936.

[B28] Horikoshi N. (2002). Occurrence of clubroot in japanese radish caused by plasmodiophora brassicae woronin in fukushima. Annu. Rep. Soc. Plant Prot. North Japan. 53, 58–60. doi: 10.11455/kitanihon1966.2002.58

[B29] Ingram D. S. Tommerup I. C. (1972). The life history of *Plasmodiophora brassicae* woron. Proc. R Soc. Lond B Biol. Sci. 18, 103–112. doi: 10.1098/rspb.1972.0008

[B30] Irani S. Todd C. D Wei Y. D Bonham-Smith P. C. (2019). Changes in phenylpropanoid pathway gene expression in roots and leaves of susceptible and resistant Brassica napus lines in response to *Plasmodiophora brassicae* inoculation. Physiol Mol Plant Pathol 106, 196–203. doi: 10.1016/j.pmpp.2019.02.007

[B31] Irani S. Trost B. Waldner M. Nayidu N. Tu J. Y. Kusalik A. J.. (2018). Transcriptome analysis of response to *Plasmodiophora brassicae* infection in the *Arabidopsis* shoot and root. BMC Genomics 19, 23. doi: 10.1186/s12864-017-4426-7 29304736 PMC5756429

[B32] Ji R. Q. Wang Y. L. Wang X. D. Liu Y. F. Shen X. Q. Feng H. (2018). Proteomic analysis of the interaction between *Plasmodiophora brassicae* and Chinese cabbage (*Brassica rapa* l. ssp. *Pekinensis*) at the initial infection stage. Sci. Hortic. 233, 386–393. doi: 10.1016/j.scienta.2018.02.006

[B33] Jorge T. F. António C. (2018). Plant metabolomics in a changing world: metabolite responses to abiotic stress combinations (London: Intech Open).

[B34] Jorge T. F. Mata A. T. António C. (2016). Mass spectrometry as a quantitative tool in plant metabolomics. Philos. Trans. A Math Phys. Eng. Sci. 374, 20150370. doi: 10.1098/rsta.2015.0370 27644967 PMC5031636

[B35] Jung S. C. Kim W. Park S. C. Jeong J. Park M. K. Lim S.. (2014). Two ginseng UDP-glycosyltransferases synthesize ginsenoside Rg3 and Rd. Plant Cell Physiol. 55 (12), 2177–2188. doi: 10.1093/pcp/pcu147 25320211

[B36] Kageyama K. Asano T. (2009). Life cycle of *Plasmodiophora brassicae* . J. Plant Growth Regul. 28, 203–211. doi: 10.1007/s00344-009-9101-z

[B37] Kamei A. Tsuro M. Kubo N. Hayashi T. Wang N. Fujimura T.. (2010). QTL mapping of clubroot resistance in radish (*Raphanus sativus* l.). Theor. Appl. Genet. 120, 1021–1027. doi: 10.1007/s00122-009-1230-z 20012934

[B38] Kim S. J. Kim A. K. (2015). Anti-breast cancer activity of fine black ginseng (*Panax ginseng* Meyer) and ginsenoside Rg5. J. Ginseng Res. 39, 125–134. doi: 10.1016/j.jgr.2014.09.003 26045685 PMC4452536

[B39] Kowata-Dresch L. S. Mio M. D. (2012). Clubroot management of highly infested soils. J. Crop Prot. 35, 47–52. doi: 10.1016/j.cropro.2011.12.012

[B40] Krumsiek J. Bartel J. Theis F. J. (2016). Computational approaches for systems metabolomics. Curr. Opin. Biotechnol. 39, 198–206. doi: 10.1016/j.copbio.2016.04.009 27135552

[B41] Laila R. Robin A. H. K. Park J. I. Saha G. Kim H. T. Kayum M. A.. (2020). Expression and role of response regulating, biosynthetic and degrading genes for cytokinin signaling during clubroot disease development. Int. J. Mol. Sci. 21, 3896–. doi: 10.3390/ijms21113896 32486099 PMC7312684

[B42] Lazebnik J. Frago E. Dicke M. Van L. Joop J. A. (2014). Phytohormone mediation of interactions between herbivores and plant pathogens. J. Chem. Ecol. 40, 730–741. doi: 10.1007/s10886-014-0480-7 25059974

[B43] Lee H. Lee S. Jeong D. Kim S. J. (2018). Ginsenoside Rh2 epigenetically regulates cell-mediated immune pathway to inhibit proliferation of MCF-7 breast cancer cells. J. Ginseng Res. 42, 455–462. doi: 10.1016/j.jgr.2017.05.003 30337805 PMC6187096

[B44] Lee O. N. Park H. Y. (2017). Assessment of genetic diversity in cultivated radishes (*Raphanus sativus*) by agronomic traits and SSR markers. Sci. Hortic. 223, 19–30. doi: 10.1016/j.scienta.2017.05.025

[B45] Lee O. N. Park H. Y. (2020). Effects of different colored film mulches on the growth and bolting time of radish *(Raphanus sativus* L.). Sci Hortic 266, 109271. doi: 10.1016/j.scienta.2020.109271

[B46] Li K. F. Kang C. M. Yin X. F. Li H. X. Chen Z. Y. Li Y.. (2018). Ginsenoside Rh2 inhibits human A172 glioma cell proliferation and induces cell cycle arrest status *via* modulating akt signaling pathway. Mol. Med. Rep. 17, 3062–3068. doi: 10.3892/mmr.2017.8193 29207171 PMC5783527

[B47] Li Y. T. Li J. X. Diao M. G. Peng L. Y. Huang S. H. Xie N. Z. (2022). Characterization of a group of udp-glycosyltransferases involved in the biosynthesis of triterpenoid saponins of *Panax notoginseng* . ACS Synth Biol. 11, 770–779. doi: 10.1021/acssynbio.1c00469 35107265

[B48] Li J. Liu T. Zhao L. Chen W. Hou H. Ye Z.. (2015). Ginsenoside 20(S)−Rg3 inhibits the warburg effect through STAT3 pathways in ovarian cancer cells. Int. J. Oncol. 46, 775–781. doi: 10.3892/ijo.2014.2767 25405516

[B49] Li L. Long Y. Li H. Wu X. (2019). Comparative transcriptome analysis reveals key pathways and hub genes in rapeseed during the early stage of *Plasmodiophora brassicae* infection. Front. Genet. 10. doi: 10.3389/fgene.2019.01275 PMC6978740 32010176

[B50] Liu L. J. Qin L. Cheng X. H. Zhang Y. Xu L. Liu F.. (2020). Comparing the infection biology of plasmodiophora brassicae in clubroot susceptible and resistant host and non-host. Front. Microbiol. 11. doi: 10.3389/fmicb.2020.507036 PMC7596292 33178139

[B51] Ludwig-Muller J. Bennett R. N. Kiddle G. Ihmig S. Ruppel M. Hilgenberg W. (1999). The host range of *Plasmodiophora brassicae* and its relationship to endogenous glucosinolate content. New Phytol. 141, 443–458. doi: 10.2307/2588412

[B52] Ludwig-Müller J. Schubert B. Pieper K. Ihmig S. Hilgenberg W. (1997). Glucosinolate content in susceptible and resistant chinese cabbage varieties during development of clubroot disease. Phytochemistry 44, 407–414. doi: 10.1016/s0031-9422(96)00498-0

[B53] Luo Y. L. Dong D. W. Su Y. Wang X. Y. Peng Y. M. Peng J.. (2018). Transcriptome analysis of *Brassica juncea* var. tumida tsen responses to *Plasmodiophora brassicae* primed by the biocontrol strain zhihengliuella aestuarii. Funct. Integr. Genomics 18, 301–314. doi: 10.1007/s10142-018-0593-0 29564648

[B54] Mao Q. Zhang P. H. Wang Q. Li S. L. (2014). Ginsenoside F2 induces apoptosis in humor gastric carcinoma cells through reactive oxygen species-mitochondria pathway and modulation of ASK-1/JNK signaling cascade *in vitro* and *in vivo* . Phytomedicine 21, 515–522. doi: 10.1016/j.phymed.2013.10.013 24252332

[B55] Mareya C. R. Tugizimana F. Di Lorenzo F. Silipo A. Piater L. A. Molinaro A.. (2020). Adaptive defence-related changes in the metabolome of sorghum bicolor cells in response to lipopolysaccharides of the pathogen burkholderia and ropogonis. Sci. Rep. 10, 7626. doi: 10.1038/s41598-020-64186-y 32376849 PMC7203242

[B56] Mohammed M. S. (2021). The molecular activity of gingerol on inhibits proliferation of breast cancer cell line (MCF7) through caspase activity. Ann. Rom Soc. Cell Biol. 25, 11095–11103.

[B57] Monson R. E. Jones K. Salmond G. P. C. (2018). Draft genome sequence of *Pectobacterium carotovorum* subsp. *carotovorum* ATCC 39048, a carbapenem-producing phytopathogen. Microbiol. Resour Announc. 7, e00825–e00818. doi: 10.1128/MRA.00825-18 30533884 PMC6256451

[B58] Moon J. Y. Kim S. T. Choi G. J. Kwon S. Y. Cho H. S. Kim H. S.. (2020). Comparative proteomic analysis of host responses to *Plasmodiophora brassicae* infection in susceptible and resistant brassica oleracea. Plant Biotechnol. Rep. 14, 263–274. doi: 10.1007/s11816-020-00596-8

[B59] Naiki T. Dixon G. R. (1987). The effects of chemicals on developmental stages of *Plasmodiophora brassicae* (clubroot). Plant Pathol. J. 36, 316–327. doi: 10.1111/j.1365-3059.1987.tb02238.x

[B60] Nicolau D. P. (2008). Carbapenems: a potent class of antibiotics. Expert Opin. Pharmacother. 9, 23–37. doi: 10.1517/14656566.9.1.23 18076336

[B61] Ning Y. Wang Y. Fang Z. Y. Zhuang M. Zhang Y. Y. Lv H. H.. (2019). Comparative transcriptome analysis of cabbage (*Brassica oleracea* var. capitata) infected by *Plasmodiophora brassicae* reveals drastic defense response at secondary infection stage. Plant Soil. 443, 167–183. doi: 10.1007/s11104-019-04196-6

[B62] Norrby S. R. (2018). Carbapenems in serious infections: a risk-benefit assessment. Drug Saf. 22, 191–194. doi: 10.2165/00002018-200022030-00003 10738843

[B63] Pedras M. S. Zheng Q. A. Strelkov S. (2008). Metabolic changes in roots of the oilseed canola infected with the biotroph *Plasmodiophora brassicae*: Phytoalexins and phytoanticipins. J. Agric. Food Chem. 56, 9949–9961. doi: 10.1021/jf802192f 18834132

[B64] Peng Y. Zhang R. Yang X. Zhang Z. Kang N. Bao L.. (2019). Ginsenoside Rg3 suppresses the proliferation of prostate cancer cell line PC3 through ROS-induced cell cycle arrest. Oncol. Lett. 17, 1139–1145. doi: 10.3892/ol.2018.9691 30655875 PMC6312957

[B65] Prerostova S. Dobrev P. Konradyova V. Knirsch V. Gaudinova A. Kramna B.. (2018). Hormonal responses to *Plasmodiophora brassicae* infection in *Brassica napus* cultivars differing in their pathogen resistance. Int. J. Mol. Sci. 19, 4024. doi: 10.3390/ijms19124024 30551560 PMC6321006

[B66] Pu Y. Pan J. Yao Y. Ngan W. Y. Yang Y. Li M.. (2021). Ecotoxicological effects of erythromycin on a multispecies biofilm model, revealed by metagenomic and metabolomic approaches. Environ. Pollut. 276, 116737. doi: 10.1016/j.envpol.2021.116737 33618119

[B67] Qian J. Li J. Jia J. G. Jin X. Qian L. Y. (2016). Ginsenoside-Rh2 inhibits proliferation and induces apoptosis of human gastric cancer sgc-7901 side population cells. Asian Pac J. Cancer Prev. 17, 1817–1821. doi: 10.7314/apjcp.2016.17.4.1817 27221858

[B68] Sanmartín N. Sánchez-Bel P. Pastor V. Pastor-Fernández J. Mateu D. Pozo M. J.. (2020). Root-to-shoot signalling in mycorrhizal tomato plants upon *Botrytis cinerea* infection. Plant Sci. 298, 110595. doi: 10.1016/j.plantsci.2020.110595 32771152

[B69] Sharma K. Gossen B. D. McDonald M. R. (2011). Effect of temperature on primary infection by *Plasmodiophora brassicae* and initiation of clubroot symptoms. Plant Pathol. 60, 830–838. doi: 10.1111/j.1365-3059.2011.02458.x

[B70] Siemens J. Keller I. Sarx J. Kunz S. Schuller A. Nagel W.. (2006). Transcriptome analysis of *Arabidopsis* clubroots indicate a key role for cytokinins in disease development. Mol. Plant Microbe Interact. 19, 480–494. doi: 10.1094/MPMI-19-0480 16673935

[B71] Silvestre W. P. Galafassi P. L. Ferreira S. D. Godinho M. Pauletti G. F. Baldasso C. (2018). Fodder radish seed cake biochar for soil amendment. Environ. Sci. pollut. Res. Int. 25, 25143–25154. doi: 10.1007/s11356-018-2571-4 29943244

[B72] Silvestre W. P. Pauletti G. F. Baldasso C. (2020). Fodder radish (*Raphanus sativus* l.) seed cake as a feedstock for pyrolysis. Ind. Crops Prod. 154, 112689. doi: 10.1016/j.indcrop.2020.112689

[B73] Song T. Chu M. G. Lahlali R. Yu F. Q. Peng G. (2016). Shotgun label-free proteomic analysis of clubroot (*Plasmodiophora brassicae*) resistance conferred by the gene rcr1 in *Brassica rapa* . Front. Plant Sci. 7. doi: 10.3389/fpls.2016.01013 PMC4939851 27462338

[B74] Song T. Lahlali R. Chu M. Karunakaran C. Peng G. (2014). “Understanding the mechanism of clubroot resistance gene *Rpb1* based on transcriptome, metabolome and Fourier transform infrared (FT-IR) analyses,” in 11th Conference of the European Foundation for Plant Pathology. Krakow, Poland.

[B75] Song T. Lahlali R. Mcgregor L. Aliferis K. A. Peng G. (2015). “Proteomic and metabolic analysis of clubroot resistance mediated by the resistance gene *Rcr1* from *Brassica rapa* ssp. chinensis,” in APS Annual Meeting. Pasadena, California.

[B76] Subbaraj A. K. Huege J. Fraser K. Cao M. S. Rasmussen S. Faville M.. (2019). A large-scale metabolomics study to harness chemical diversity and explore biochemical mechanisms in ryegrass. Commun. Biol. 2, 87. doi: 10.1038/s42003-019-0289-6 30854479 PMC6399292

[B77] Tang H. Li P. Chen P. Ma J. K. Guo H. H. Huang X. C.. (2022). The formation mechanisms of key flavor substances in stinky tofu brine based on metabolism of aromatic amino acids. Food Chem. 392, 133253. doi: 10.1016/j.foodchem.2022.133253 35649310

[B78] Wang L. J. He J. C. Wang L. F. Gu Y. W. Tian H. J. (2020). Neuroprotective effect of ginsenoside Rb-1 on a rat model of alzheimer’s disease. Zhonghua Yi Xue Za Zhi. 100, 2462–2466. doi: 10.3760/cma.j.cn112137-202000123-00151 32819064

[B79] Wang J. J. Tian L. Khan M. N. Zhang L. Chen Q. Zhao Y.. (2018). Ginsenoside Rg3 sensitizes hypoxic lung cancer cells to cisplatin *via* blocking of NF-κB mediated epithelial–mesenchymal transition and stemness. Cancer Lett. 415, 73–85. doi: 10.1016/j.canlet.2017.11.037 29199005

[B80] Wei X. C. Xu W. Yuan Y. X. Yao Q. J. Zhao Y. Y. Wang Z. Y.. (2016). Genome-wide investigation of microRNAs and their targets in brassica rapa ssp. *pekinensis* root with *Plasmodiophora brassicae* infection. Hortic. Plant J. 2, 209–216. doi: 10.1016/j.hpj.2016.11.004

[B81] Xue P. Yang X. S. Zhao L. Hou Z. H. Zhang R. Y. Zhang F. X.. (2020). Relationship between antimicrobial activity and amphipathic structure of ginsenosides. Ind. Crops Prod. 143, 111929. doi: 10.1016/j.indcrop.2019.111929

[B82] Xu J. Pan Y. Liu Y. Na S. Zhou H. Li L.. (2021). A review of anti-tumour effects of ginsenoside in gastrointestinal cancer. J. Pharm. Pharmacol. 73, 1292–1301. doi: 10.1093/jpp/rgab048 33836068

[B83] Yang H. Zheng J. Fu Y. D. Zhang Y. H. Yi C. L. Jin C.. (2020). Specific genes and sequence variation in pathotype 7 of the clubroot pathogen *Plasmodiophora brassicae* . Eur. J. Plant Pathol. 1, 1–12. doi: 10.1007/s10658-020-01968-0

[B84] Yan H. Jin H. Fu Y. Yin Z. Yin C. (2019). Production of rare ginsenosides Rg3 and Rh2 by endophytic bacteria from *Panax ginseng* . J. Agric. Food Chem. 67 (31), 8493–8499. doi: 10.1021/acs.jafc.9b03159 31310523

[B85] Yusof K. M. Makpol S. Fen L. S. Jamal R. Ngah W. Z. W. (2021). Suppression of colorectal cancer cell growth by combined treatment of 6-gingerol and γ-tocotrienol *via* alteration of multiple signalling pathways. J. Nat. Med. 73, 745–760. doi: 10.1007/s11418-019-01323-6 31177355

[B86] Yu R. G. Wang J. Xu L. Wang Y. Wang R. H. Zhu X. W.. (2016b). Transcriptome profiling of taproot reveals complex regulatory networks during taproot thickening in radish (*Raphanus sativus* l.). Front. Plant Sci. 7. doi: 10.3389/fpls.2016.01210 PMC4992731 27597853

[B87] Yu R. G. Xu L. Zhang W. Wang Y. Luo X. B. Wang R. H.. (2016a). *De novo* taproot transcriptome tequencing and analysis of major genes involved in sucrose metabolism in radish (*Raphanus sativus* l.). Front. Plant Sci. 7. doi: 10.3389/fpls.2016.00585 PMC4868836 27242808

[B88] Zamani-Noor N. Hornbacher J. Comel C. J. Papenbrock J. (2021). Variation of glucosinolate contents in clubroot-resistant and -susceptible *Brassica napus* cultivars in response to virulence of plasmodiophora brassicae. Pathogens 10, 563. doi: 10.3390/pathogens10050563 34066620 PMC8148440

[B89] Zhang X. Liu Y. Fang Z. Li Z. Yang L. Zhuang M.. (2016). Comparative transcriptome analysis between broccoli (*Brassica oleracea* var. italica) and wild cabbage (*Brassica macrocarpa* guss.) in response to *Plasmodiophora brassicae* during different infection stages. Front. Plant Sci. 7. doi: 10.3389/fpls.2016.01929 PMC5179516 28066482

[B90] Zhao Y. Bi K. Gao Z. X. Chen T. Liu H. Q. Xie J. T.. (2017). Transcriptome analysis of *Arabidopsis* thaliana in response to *Plasmodiophora brassicae* during early infection. Front. Microbiol. 8. doi: 10.3389/fmicb.2017.00673 PMC5401899 28484434

[B91] Zhou P. Xie W. J. He S. B. Sun Y. F. Meng X. B. Sun G. B.. (2019). Ginsenoside Rb1 as an anti-diabetic agent and its underlying mechanism analysis. Cells 8, 204. doi: 10.3390/cells8030204 30823412 PMC6468558

[B92] Zhou Y. Y. Zheng X. Lu J. J. Chen W. Li X. Zhao L. (2018). Ginsenoside 20(S)-Rg3 inhibits the warburg effect *via* modulating DNMT3A/MiR-532-3p/HK2 pathway in ovarian cancer cells. Cell. Physiol. Biochem. 45, 2548–2559. doi: 10.1159/000488273 29558748

[B93] Zhuang J. Yin J. Xu C. Mu Y. Lv S. (2018). 0(S)-Ginsenoside Rh2 Induce the Apoptosis and Autophagy in U937 and K562 Cells. Nutrients 10, 328. doi: 10.3390/nu10030328 29518056 PMC5872746

