# Supplementary material for: Metabonomic profiling of clubroot-susceptible and clubroot-resistant radish and the assessment of disease-resistant metabolites
Source: Front Plant Sci. 2022 Dec 8;13:1037633. doi: 10.3389/fpls.2022.1037633 (PMC9772615; doi:10.3389/fpls.2022.1037633)
Supplement: Supplementary file 1 [file DataSheet_1.zip › NEW Frontiers Supplementary Material/frontiers supplementary Table 1.docx]

| Supplementary Table 1. Clubroot disease incidence and index of radish cultivars grown in *P. brassicae*–contaminated radish farm in Weining county, Bijie City, Guizhou province, China. | | | | | | | | | | | | |
| --- | --- | --- | --- | --- | --- | --- | --- | --- | --- | --- | --- | --- |
| Disease resistance | | | Disease Tolerance | | | Disease Susceptible | | | Disease High susceptibility | | | |
| Cultivar | Disease incidence | Disease index | Cultivar | Disease incidence | Disease index | Cultivar | Disease incidence | Disease index | Cultivar | | Disease incidence | Disease index |
| Daehanbaekchun | 6.67% | 0.74 | Chunbaiyu | 33.33% | 11.12 | Xiuyu F1 | 100.00% | 33.33 | Bingtang radish | | 76.67% | 62.59 |
| Morisaki radish | 23.33% | 2.59 | Weixian radish | 56.67% | 11.48 | Chuitianqingtou fruit radish | 86.67% | 33.33 | Daqingtou radish | | 100.00% | 65.19 |
| Hanxue radish | 26.67% | 2.96 | Gaoyuanqiu white radish | 36.67% | 11.48 | Liangpincuilv | 70.00% | 35.93 | Jinke fruit radish | 100.00% | | 73.33 |
| Xinbaijngyu | 23.33% | 3.33 | Sijimanshenhong | 43.33% | 11.48 | Han cui | 80.00% | 37.04 | Dachangqingtou | | 100.00% | 77.78 |
| Baiyu No.1 | 30.00% | 3.33 | Hanbaiyu | 36.67% | 11.48 | Shinongdimachun | 76.67% | 38.15 | Degaoshengdin radish | | 100.00% | 88.89 |
| Chunxuelian | 20.00% | 3.70 | Nanpanzhou tebiezhong | 56.67% | 11.48 | Chang qingtou | 100.00% | 38.52 | Jiangnanyuanbai | | 100.00% | 98.89 |
| Xinjiangjun | 23.33% | 4.07 | Teji baiJinyu | 30.00% | 12.22 | Jiangshugailiang  Weixian green radish | 96.67% | 38.89 |  | |  |  |
| Xinxuan manshenhong | 36.67% | 4.07 | Dongxue | 76.67% | 12.96 | Deri NO 2 | 96.67% | 38.89 |  | |  |  |
| Jiangjun | 26.67% | 4.44 | Xinzhedachang | 50.00% | 12.96 | Hybridbanye nantanghong F1 | 93.33% | 45.93 |  | |  |  |
| Texin baiyuchun | 26.67% | 4.44 | Jingyanxueyu | 50.00% | 12.96 | Flanders NO 5 | 93.33% | 50.37 |  | |  |  |
| Jingdianduanhongpi | 40.00% | 4.44 | Jiujingwang | 73.33% | 13.33 |  |  |  |  | |  |  |
| Chunbaiyu | 30.00% | 4.81 | Xinxueliang | 63.33% | 13.70 |  |  |  |  | |  |  |
| Zhongluo No.1 | 46.67% | 5.19 | PingyouCuiyu radish | 63.33% | 14.44 |  |  |  |  | |  |  |
| Baiyuchun | 23.33% | 5.56 | Jinghong NO 4 F1 | 43.33% | 14.44 |  |  |  |  | |  |  |
| Jieyou | 46.67% | 5.93 | Chuitian touxinhong | 43.33% | 14.44 |  |  |  |  | |  |  |
| Chuanhe baiyu | 33.33% | 5.93 | Baiyu NO 2 | 46.67% | 14.81 |  |  |  |  | |  |  |
| Hanxiang | 26.67% | 5.93 | Cuiyu radish | 50.00% | 15.19 |  |  |  |  | |  |  |
| Yachun No.1 | 40.00% | 5.93 | Hanyuanbaiyu | 70.00% | 15.19 |  |  |  |  | |  |  |
| Baichuanrongyao | 20.00% | 5.93 | Tianzinanpanzhou wanluobo | 46.67% | 15.56 |  |  |  |  | |  |  |
| Hanjiang xueliang | 63.33% | 7.04 | ZhongluoNO1radish | 46.67% | 15.56 |  |  |  |  | |  |  |
| Han bairuyu | 36.67% | 7.04 | Hybrid F1 | 70.00% | 15.93 |  |  |  |  | |  |  |
| Hanchengxue | 33.33% | 7.04 | 791 Radish | 56.67% | 15.93 |  |  |  |  | |  |  |
| Teji baijinyu | 43.33% | 7.04 | Teda shaguangqingtou | 50.00% | 15.93 |  |  |  |  | |  |  |
| Oriental baixue | 36.67% | 7.04 | Texinjiangjun | 70.00% | 15.93 |  |  |  |  | |  |  |
| DongchunA1 radish | 30.00% | 7.04 | Chunnong NO 6 | 36.67% | 15.93 |  |  |  |  | |  |  |
| YRxinbaiyuchun | 33.33% | 7.41 | Deshuqiuyu 791 | 46.67% | 16.30 |  |  |  |  | |  |  |
| Shijichun radish | 30.00% | 7.78 | Dongchunyu radish | 76.67% | 16.67 |  |  |  |  | |  |  |
| Jiguang | 23.33% | 7.78 | Pixian heiyechunbulao | 53.33% | 17.78 |  |  |  |  | |  |  |
| Hanjiangdageng | 30.00% | 7.78 | Luotouqing radish | 53.33% | 17.78 |  |  |  |  | |  |  |
| Xue wang chun | 30.00% | 8.52 | Seoul xueyu | 60.00% | 17.78 |  |  |  |  | |  |  |
| South Korea bai yun | 53.33% | 8.89 | Dabang wanluobo | 76.67% | 18.15 |  |  |  |  | |  |  |
| GY- Belle | 26.67% | 8.89 | Degao daqing | 70.00% | 18.89 |  |  |  |  | |  |  |
| Baiyu No.1 | 53.33% | 10.37 | Zhongda 791 | 73.33% | 20.00 |  |  |  |  | |  |  |
| Techanggangmiluobo | 40.00% | 10.37 | Nanpanzhou radish | 60.00% | 20.00 |  |  |  |  | |  |  |
| Beijingxinlimei | 76.67% | 10.74 | Bairuyu radish | 46.67% | 20.00 |  |  |  |  | |  |  |
|  |  |  | Hanjinbaiyu | 73.33% | 20.74 |  |  |  |  | |  |  |
|  |  |  | Chuibao radish | 83.33% | 24.07 |  |  |  |  | |  |  |
|  |  |  | Pinyou NO 4 | 63.33% | 24.07 |  |  |  |  | |  |  |
|  |  |  | Xinfengguang F1 | 73.33% | 24.44 |  |  |  |  | |  |  |
|  |  |  | Baishananpanzhouwanluobo | 80.00% | 24.44 |  |  |  |  | |  |  |
|  |  |  | Liangpinxiuli | 90.00% | 24.81 |  |  |  |  | |  |  |
|  |  |  | Qingyu radish | 76.67% | 25.56 |  |  |  |  | |  |  |
|  |  |  | Degaoqingquan | 86.67% | 28.89 |  |  |  |  | |  |  |
|  |  |  | Liang guang | 93.33% | 31.11 |  |  |  |  | |  |  |
|  |  |  | Naichoutai luobo | 76.67% | 32.96 |  |  |  |  | |  |  |
|  |  |  | Yuxingcuixue | 76.67% | 32.96 |  |  |  |  | |  |  |

Note: A total of three experimental plots, each plot has 20 plants of each cultivar, a total of 97 cultivars. Data are presented by mean value.
