# Supplementary material for: Metabonomic profiling of clubroot-susceptible and clubroot-resistant radish and the assessment of disease-resistant metabolites
Source: Front Plant Sci. 2022 Dec 8;13:1037633. doi: 10.3389/fpls.2022.1037633 (PMC9772615; doi:10.3389/fpls.2022.1037633)
Supplement: Supplementary file 1 [file DataSheet_1.zip › NEW Frontiers Supplementary Material/frontiers supplementary Table 3.docx]

Supplementary Table 3. DRMs that significantly regulated both in “JNYB” and “DHBC”.

| ID | Regulated (“JNYB”, “DHBC”) | VIP (“JNYB”, “DHBC”) | MS2Metabolite | MS2superclass | t.test_p.value  (“JNYB”, “DHBC”) |
| --- | --- | --- | --- | --- | --- |
| M566T390 | up,  down | 2.812168, 4.161531 | LysoPC 18:1 | Lipids and lipid-like molecules | 5.73E-10,  1.86E-06 |
| M564T369 | up,  down | 2.603168, 3.355031 | LysoPC 18:2 | Lipids and lipid-like molecules | 4.33E-06,  3.25E-05 |
| M478T386 | up,  down | 2.542168, 4.624431 | LysoPE 18:1 | Lipids and lipid-like molecules | 1.03E-10,  5.16E-06 |
| M478T366 | up,  down | 2.506168, 3.656731 | LysoPE 18:2 | Lipids and lipid-like molecules | 4.43E-11,  8.99E-05 |
| M452T383 | up,  down | 2.417168, 3.806031 | LysoPE 16:0 | Lipids and lipid-like molecules | 1.23E-06, 0.000244 |
| M617T251 | down,  down | 1.896168, 2.842631 | - | Unknown | 2.03E-09,  0.019814 |
| M562T353 | up,  down | 1.879168, 3.899131 | LysoPC 18:3 | Lipids and lipid-like molecules | 1.335E-07,  0.000282 |
| M496T387 | up,  down | 1.742168, 3.762731 | LysoPC 16:0 | Lipids and lipid-like molecules | 0.035370,  1.01E-05 |
| M521T427 | up,  down | 1.657168, 2.660931 | - | Unknown | 9.93E-07,  0.000342 |
| M561T212 | down,  up | 1.568168, 2.520331 | - | Unknown | 0.035370,  0.035705 |
| M287T355 | down,  down | 1.477168, 2.890431 | - | Unknown | 4.83E-05, 0.003504 |
| M476T351_1 | up,  down | 1.424168, 4.840631 | LysoPE 18:3 | Lipids and lipid-like molecules | 2.43E-09, 0.000144 |
| M362T203 | down,  up | 1.3181686, 2.014031 | - | Unknown | 1.83E-07, 0.027560 |
| M229T43 | down,  down | 1.193168, 2.705331 | - | Unknown | 0.035370, 0.006084 |
| M425T166 | up,  down | 1.106168, 2.375831 | - | Unknown | 2.03E-05, 0.026297 |
