# Supplementary material for: Metabonomic profiling of clubroot-susceptible and clubroot-resistant radish and the assessment of disease-resistant metabolites
Source: Front Plant Sci. 2022 Dec 8;13:1037633. doi: 10.3389/fpls.2022.1037633 (PMC9772615; doi:10.3389/fpls.2022.1037633)
Supplement: Supplementary file 1 [file DataSheet_1.zip › NEW Frontiers Supplementary Material/Frontiers Supplementary Figure.docx]

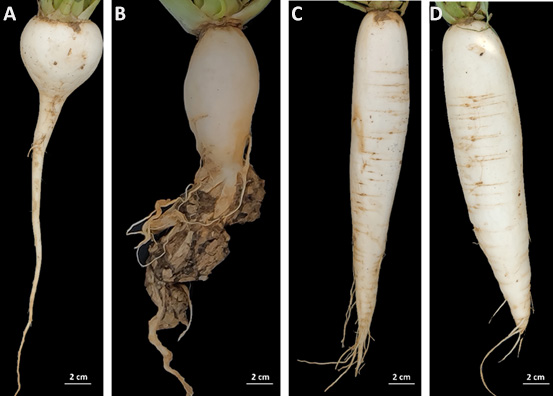


**Supplementary Figure 1.** Features of healthy(A) , clubroot diseased “JNYB”(B), healthy “DHBC” **(C)** and infected “DHBC” **(D)** grown in *P. brassicae* contaminated field.


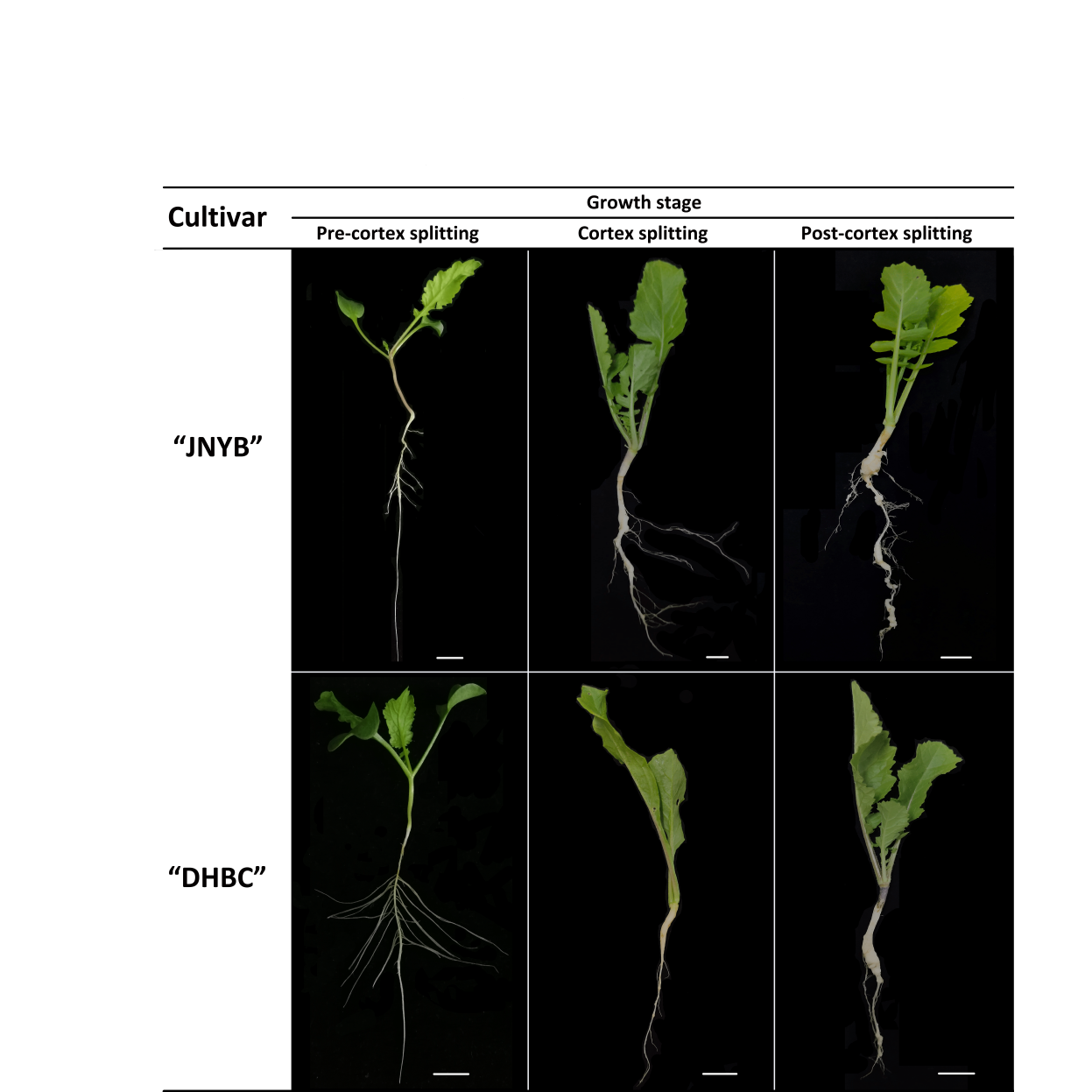


**Supplementary Figure 2.** Phenotypes of two radish cultivar in per-cortex splitting, cortex splitting, and post-cortex splitting after inoculation with resting spores.


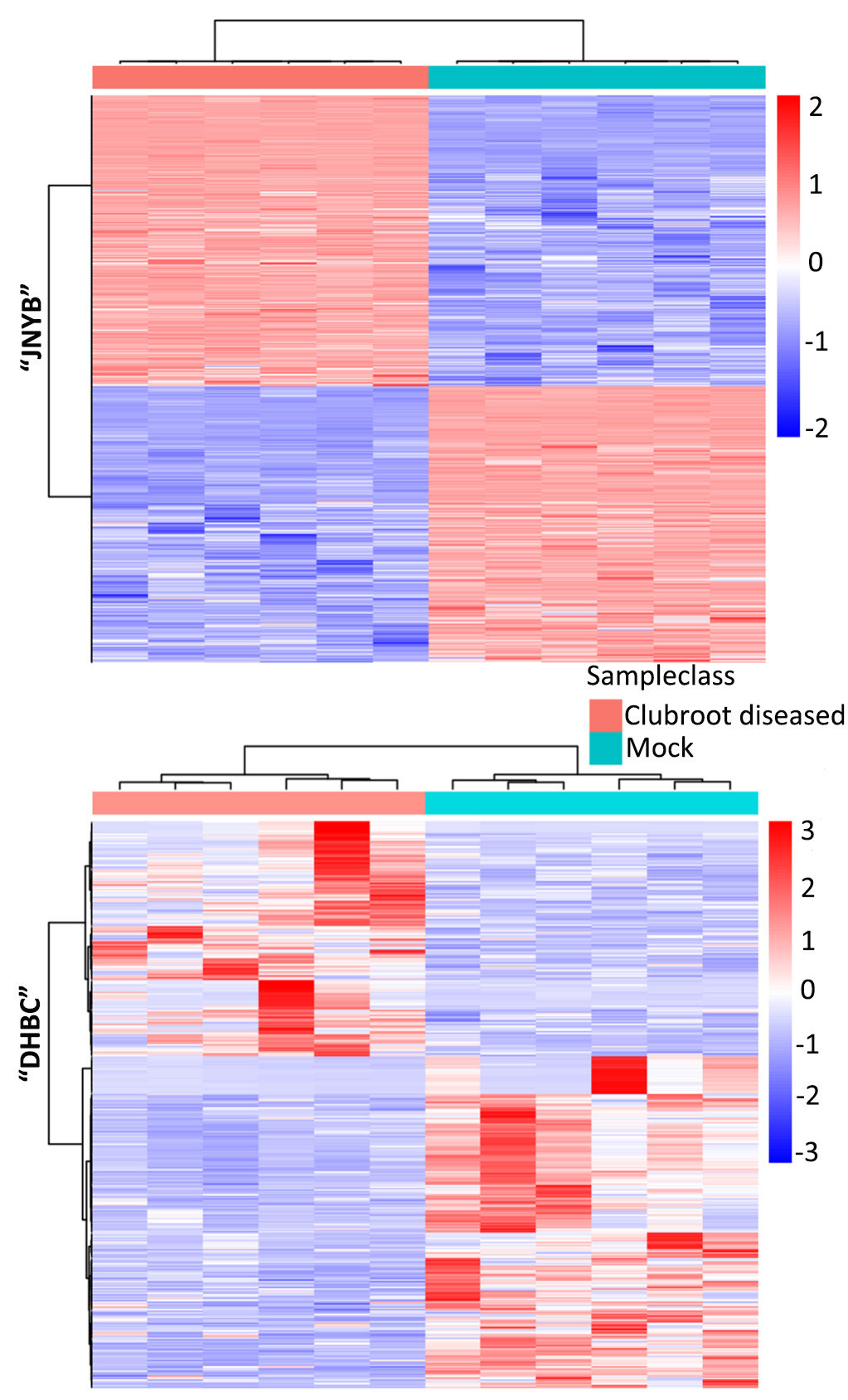


**Supplementary Figure 3.** Expression patterns of the different regulated metabolites between “JNYB” and “DHBC” under *P. brassicae* infection. The heatmap presents normalized FPKM expression values. “JNYB” is clubroot susceptible cultivar, “DHBC” is the clubroot resistant cultivar.


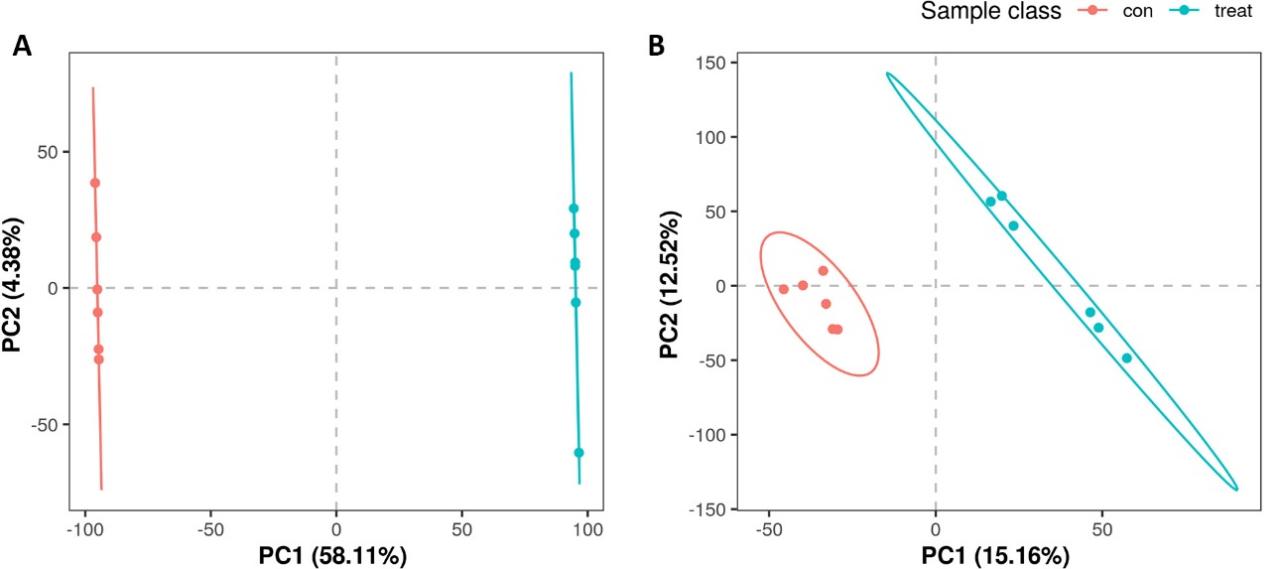


**Supplementary Figure 4.** Partial least squares-discriminant analysis (PLS-DA) of metabolic profiles of “DHBC” (A) and “JNYB” (B) under control and *P. brassicae* infection.Con equals to control; treat equals to diseased samples.


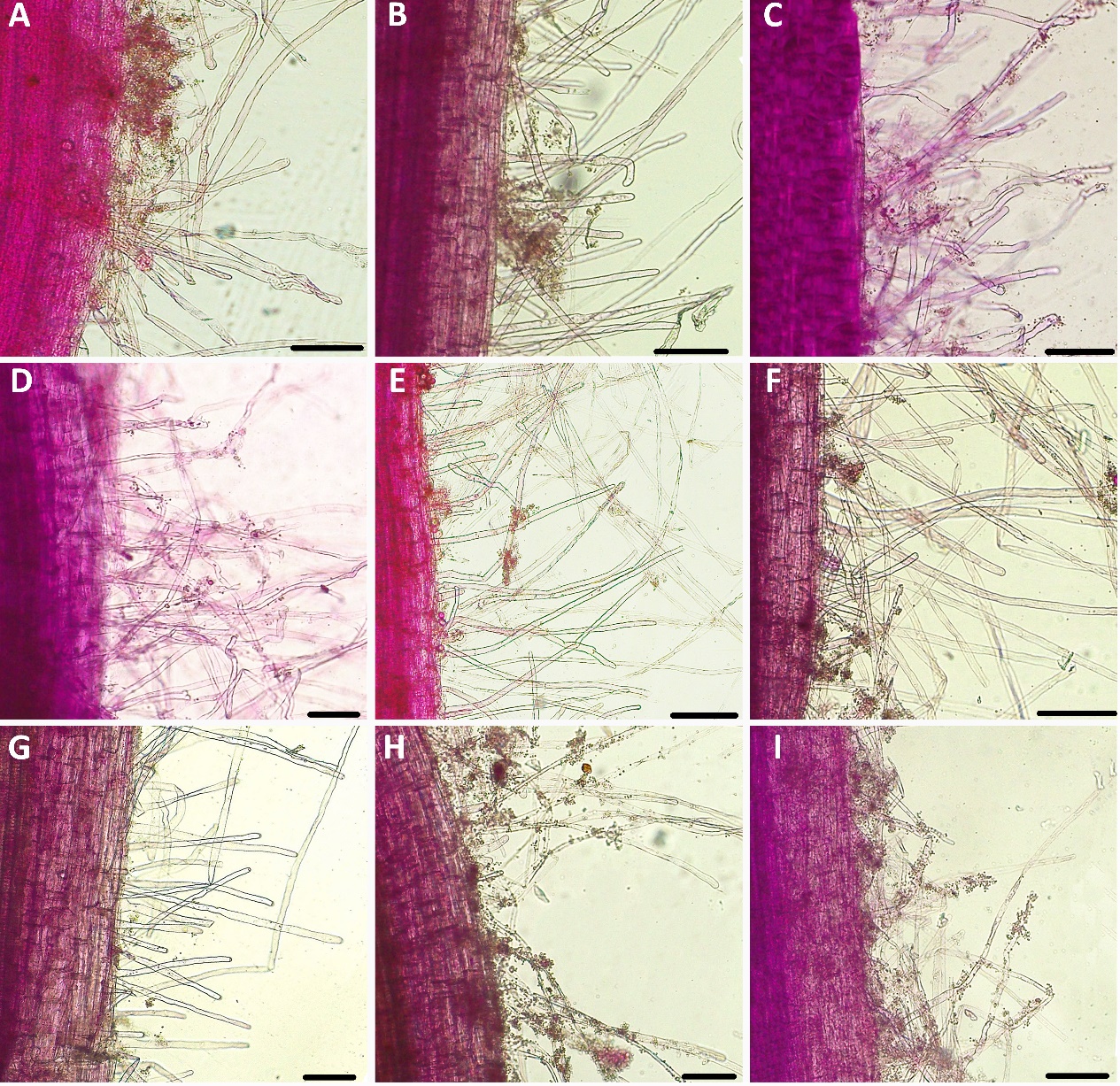


**Supplementary Figure 5.** The effects of metabolite used alone on the enrichment of *P. brassicae* around root hair of radish photographed 24 h post-inoculation with resting spores. **(A)** indicates mock, **(B)** and **(C)** refers to 2.5 mg/L and 5 mg/L ethyl α-D-Thioglucopyranoside treatments; **(D)** and **(E)** refers to 10 mg/L and 20 mg/L of imipenem treatments; **(F)** and **(G)** indicates the effects of 2.5 mg/L and 5 mg/L ginsenoside treatments; **(H)** and **(I)** refers to 2.5 mg/L and 5 mg/L 6-Gingerol treatments. Scale bar equals to 100 μm.


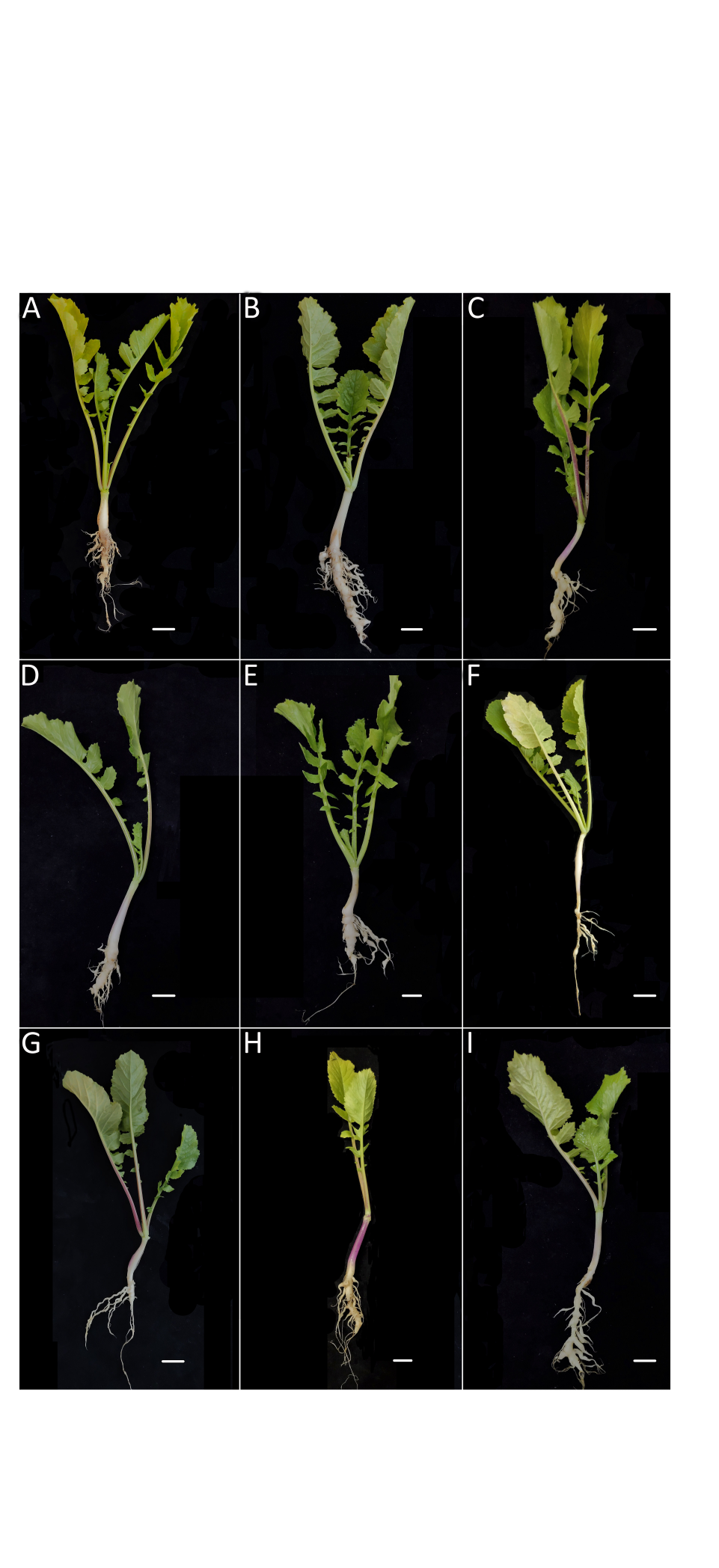


**Supplementary Figure 6.** The effects of metabolite used alone on the clubroot disease severity in radish tap root photographed at the stage of cortex splitting. **(A)** indicates mock, **(B)** and **(C)** refers to 2.5 mg/L and 5 mg/L ethyl α-D-Thioglucopyranoside treatments; **(D)** and **(E)** refers to 10 mg/L and 20 mg/L of imipenem treatments; **(F)** and **(G)** indicates the effects of 2.5 mg/L and 5 mg/L ginsenoside treatments; **(H)** and **(I)** refers to 2.5 mg/L and 5 mg/L 6-Gingerol treatments. Scale bar equals to 1 cm.


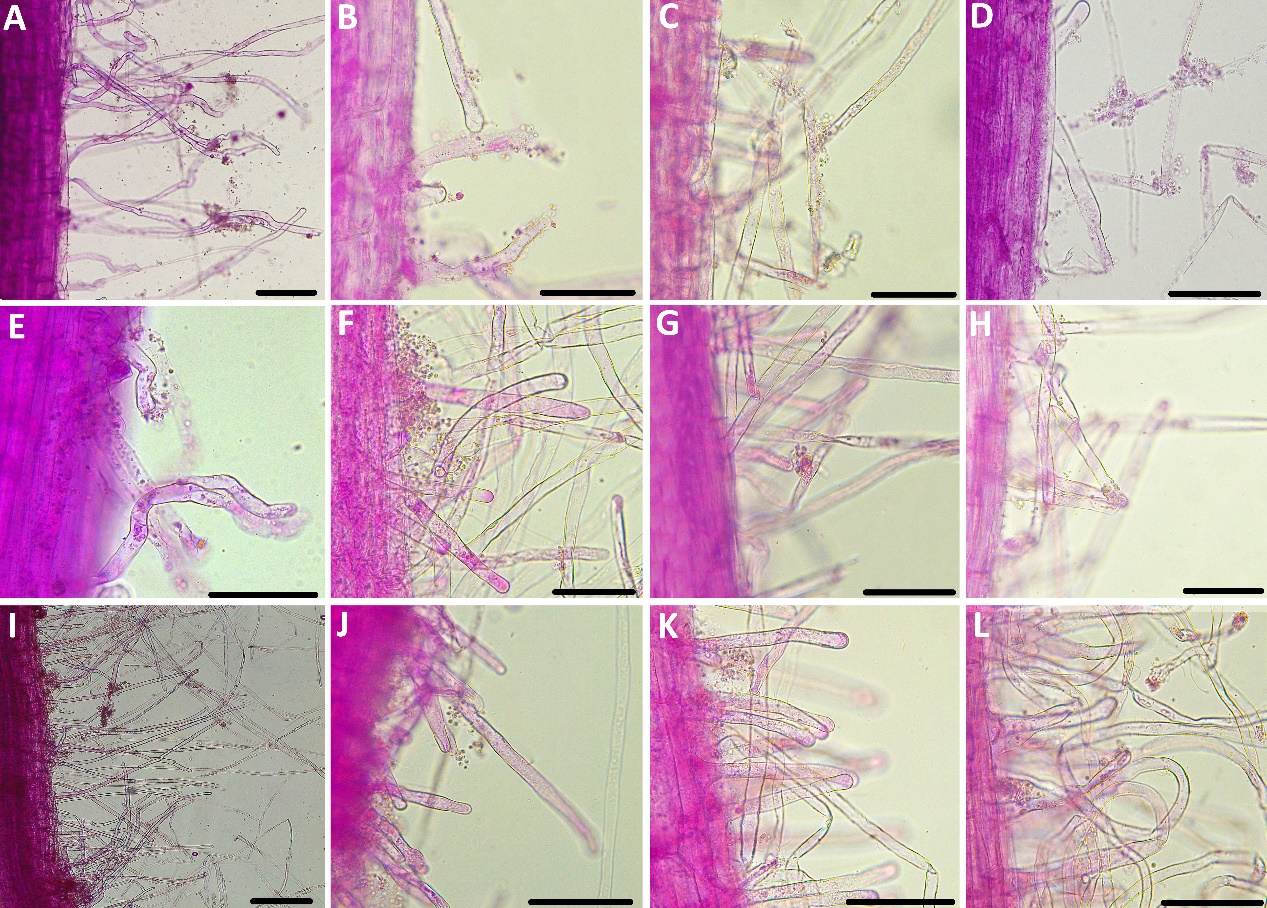


**Supplementary Figure 7.** The effects of metabolite combinations on the enrichment of *P. brassicae* around root hair of radish photographed 24 h post-inoculation with resting spores. **(A)** indicates mock, **(B-G)** refers to 5 mg/L Ethyl α-D-Thioglucopyranoside + 20 mg/L imipenem treatment, 5 mg/L Ethyl α-D-Thioglucopyranoside + 5 mg/L ginsenoside, 5 mg/L Ethyl α-D-Thioglucopyranoside + 5mg/L 6-gingerol treatment, 20 mg/L imipenem +5 mg/L ginsenoside, 20 mg/L imipenem + 5 mg/L 6-gingerol, 5 mg/L ginsenoside + 5 mg/L 6-gingerol; **(H-K)** refers to 5 mg/L Ethyl α-D-Thioglucopyranoside + 20 mg/L imipenem + 5 mg/L ginsenoside, 5 mg/L Ethyl α-D-Thioglucopyranoside + 20 mg/L imipenem + 5 mg/L 6-gingerol, 5 mg/L Ethyl α-D-Thioglucopyranoside + 5 mg/L ginsenoside + 5 mg/L 6-gingerol and 20 mg/L imipenem + 5 mg/L ginsenoside + 5 mg/L 6-gingerol treatments; **(L)** refers to 5 mg/L Ethyl α-D-Thioglucopyranoside + 20 mg/L imipenem + 5 mg/L ginsenoside + 5 mg/L 6-gingerol. Scale bar equals to 100 μm.


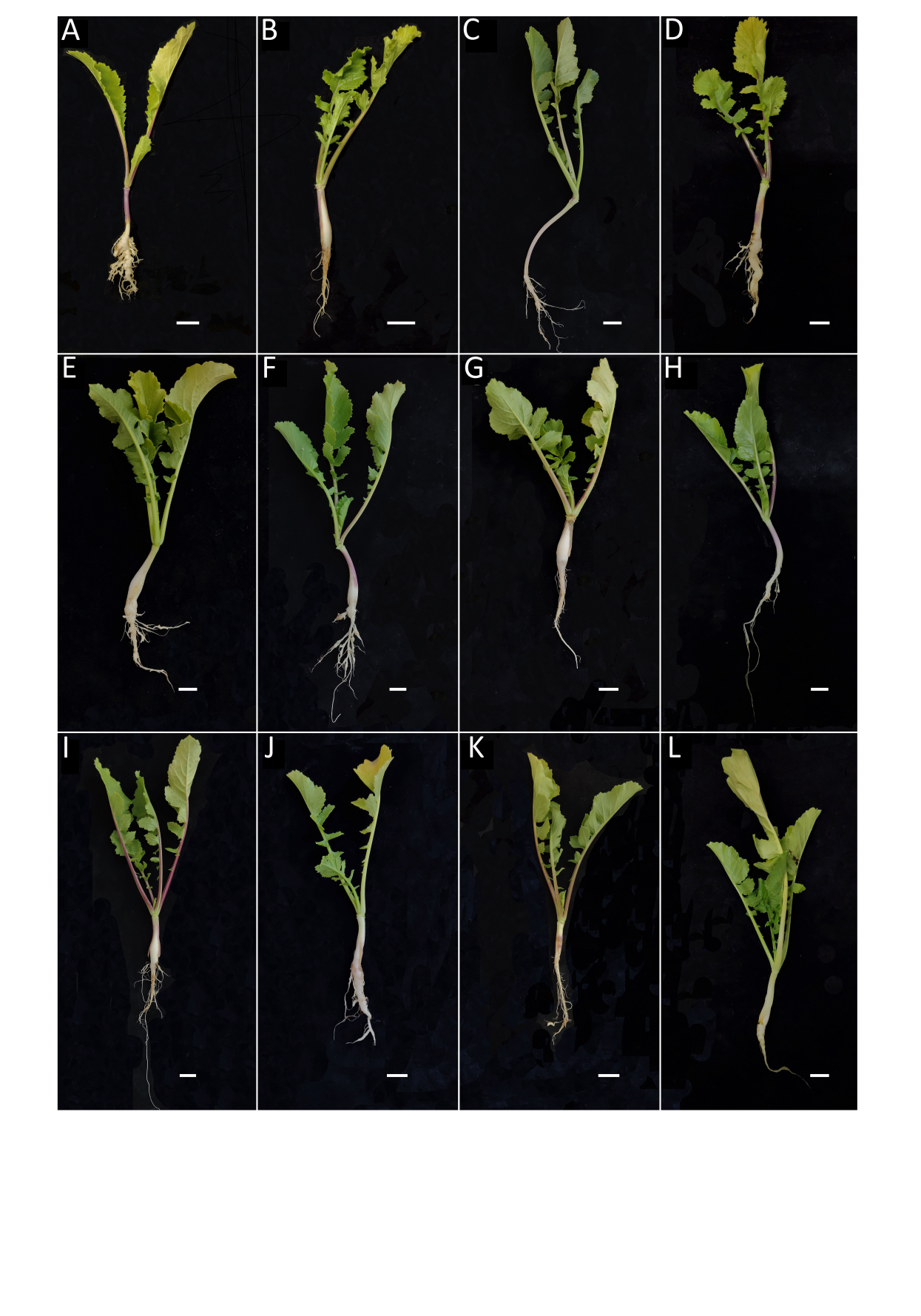


**Supplementary Figure 8.** The effects of metabolite combinations on the clubroot disease severity in radish tap root photographed at the stage of cortex splitting. **(A)** indicates mock, **(B-G)** refers to 5 mg/L Ethyl α-D-Thioglucopyranoside + 20 mg/L imipenem treatment, 5 mg/L Ethyl α-D-Thioglucopyranoside + 5 mg/L ginsenoside, 5 mg/L Ethyl α-D-Thioglucopyranoside + 5 mg/L 6-gingerol treatment, 20 mg/L imipenem +5 mg/L ginsenoside, 20 mg/L imipenem + 5 mg/L 6-gingerol, 5 mg/L ginsenoside + 5 mg/L 6-gingerol; **(H-K)** refers to 5 mg/L Ethyl α-D-Thioglucopyranoside + 20 mg/L imipenem+5 mg/L ginsenoside, 5 mg/L Ethyl α-D-Thioglucopyranoside + 20 mg/L imipenem +5 mg/L 6-gingerol, 5 mg/L Ethyl α-D-Thioglucopyranoside + 5 mg/L ginsenoside + 5 mg/L 6-gingerol and 20 mg/L imipenem + 5 mg/L ginsenoside + 5 mg/L 6-gingerol treatments; **(L)** refers to 5 mg/L Ethyl α-D-Thioglucopyranoside + 20 mg/L imipenem + 5 mg/L ginsenoside + 5 mg/L 6-gingerol. Scale bar equals to 100 μm.
